# ﻿Species diversity and major host/substrate associations of the genus *Akanthomyces* (Hypocreales, Cordycipitaceae)

**DOI:** 10.3897/mycokeys.101.109751

**Published:** 2024-01-15

**Authors:** Yao Wang, Zhi-Qin Wang, Run Luo, Sisommay Souvanhnachit, Chinnapan Thanarut, Van-Minh Dao, Hong Yu

**Affiliations:** 1 Yunnan Herbal Laboratory, College of Ecology and Environmental Sciences, Yunnan University, Kunming, Yunnan, China; 2 The International Joint Research Center for Sustainable Utilization of Cordyceps Bioresources in China and Southeast Asia, Yunnan University, Kunming, Yunnan, China; 3 Faculty of Agricultural Production, Maejo University, Chiang Mai, Thailand; 4 Institute of Regional Research and Development, Ministry of Science and Technology, Hanoi, Vietnam

**Keywords:** Arthropod-pathogenic fungi, Cordycipitaceae, morphology, new species, phylogenetic analyses

## Abstract

*Akanthomyces*, a group of fungi with rich morphological and ecological diversity in Cordycipitaceae (Ascomycota, Hypocreales), has a wide distribution amongst diverse habitats. By surveying arthropod-pathogenic fungi in China and Southeast Asia over the last six years, nine *Akanthomyces* spp. were found and identified. Five of these were shown to represent four known species and an undetermined species of *Akanthomyces*. Four of these were new species and they were named *A.kunmingensis* and *A.subaraneicola* from China, *A.laosensis* from Laos and *A.pseudonoctuidarum* from Thailand. The new species were described and illustrated according to the morphological characteristics and molecular data. *Akanthomycesaraneogenus*, which was isolated from spiders from different regions in China, Thailand and Vietnam, was described as a newly-recorded species from Thailand and Vietnam. The phylogenetic positions of the nine species were evaluated, based on phylogenetic inferences according to five loci, namely, ITS, nrLSU, *TEF*, *RPB1* and *RPB2*. In this study, we reviewed the research progress achieved for *Akanthomyces* regarding its taxonomy, species diversity, geographic distribution and major host/substrate associations. The morphological characteristics of 35 species in *Akanthomyces*, including four novel species and 31 known taxa, were also compared.

## ﻿Introduction

*Akanthomyces* Lebert is one of the oldest genera in the family Cordycipitaceae (Ascomycota, Hypocreales). This genus was established by Lebert in 1858 on the basis of the type species, *A.aculeatus* Lebert, which was found on a moth in France (Lebert 1858). Morphologically, *Akanthomyces* species have been characterised asexually by white, cream or flesh-coloured cylindrical, attenuated synnematal growth covered by a hymenium-like layer of phialides producing one‐celled catenulate conidia (Mains 1950; Samson and Evans 1974; Hsieh et al. 1997). These phialides are ellipsoidal, cylindrical or narrowly cylindrical and gradually or abruptly taper to a more or less distinct neck (Hsieh et al. 1997). Owing to extensive overlap in their morphological characteristics, *Akanthomyces* was once considered as a synonym of *Lecanicillium* W. Gams & Zare, an anamorph within Cordycipitaceae with verticillium-like morphology (Gams and Zare 2001); however, many species originally described in *Lecanicillium* do not form a single monophyletic clade and are distributed throughout Cordycipitaceae (Wang et al. 2020). Kepler et al. (2017) phylogenetically established the genetic boundaries in Cordycipitaceae and they proposed that *Lecanicillium* should be rejected and, instead, could be considered as a synonym of *Akanthomyces* (Kepler et al. 2017). Kepler et al. (2017) also showed that the type species of *Lecanicillium*, *L.lecanii* (Zimm.) Zare & W. Gams (as *Cordycepsconfragosa* (Mains) G.H. Sung, J.M. Sung, Hywel-Jones & Spatafora), as well as several other *Lecanicillium* species, namely, *L.attenuatum* Zare & W. Gams, *L.muscarium* (Petch) Zare & W. Gams and *L.sabanense* Chir.-Salom., S. Restrepo & T.I. Sanjuan, fall within *Akanthomyces*. The teleomorph of *Akanthomyces* was originally described as *Torrubiella* Boud. and it was characterised by producing superficial perithecia on a loose mat of hyphae (subiculum) or a highly reduced non-stipitate stroma (Boudier 1885). According to the most complete taxonomic treatment of Cordycipitaceae to date, this connection was verified by DNA sequencing; since *Akanthomyces* was described earlier than *Torrubiella*, the taxonomic revision recommended *Akanthomyces* as the name of this genus (Kepler et al. 2017).

Over the past two decades, our efforts have been applied to the investigation of Cordycipitoid fungi, especially those located in China and Southeast Asia. To date, our study team has collected over 18,000 specimens and 7,500 strains of *Cordyceps* Fr. *sensu lato*, representing more than 450 species in total (Wang et al. 2020). These specimens and strains sufficiently revealed that Cordycipitaceae is the most complex group in Hypocreales with its varied morphological characteristics and wide-ranging hosts. Some of the genera with sexual and asexual morphs, such as *Akanthomyces* and *Hevansia* Luangsa-ard, Hywel-Jones & Spatafora, share numerous similar morphological characteristics. The genus *Hevansia* was erected to accommodate asexual morphs on spiders that were previously described under *Akanthomyces*. The type species *Hevansianovoguineensis* (Samson & B.L. Brady) Luangsa-ard, Hywel-Jones & Spatafora, which was previously described as *Akanthomycesnovoguineensis* Samson & B.L. Brady, differs from *Akanthomyces* by the immersed perithecia of the teleomorph in a disc sitting at the top of a well-formed stipe (Aini et al. 2020); however, *H.novoguineensis* must now be an akanthomyces-like teleomorph (Kepler et al. 2017; Aini et al. 2020). Some *Akanthomyces*, *Samsoniella* Mongkols., Noisrip., Thanakitp., Spatafora & Luangsa-ard and *Cordyceps* species produce similar isaria-like asexual conidiogenous structures, such as flask-shaped phialides produced in whorls and conidia with divergent chains (Wang et al. 2020; Wang et al. 2022). Due to the extensive overlap in morphological characteristics and the lack of distinctive phenotypic variation, species in many genera, *Akanthomyces* in particular, are not easily classified and identified. Thus, more known species and new species in the genus *Akanthomyces* need to be introduced and supported by more detailed morphological and phylogenetic evidence in combination with a larger taxon sampling.

In surveys of arthropod-pathogenic fungi from different regions in Yunnan and Hunan Province, China; Chiang Mai Province, Thailand; Nghe An Province, Vietnam; and Oudomxay Province, Laos, over the last six years, approximately nine *Akanthomyces* spp. were collected and identified. In this study, we aimed to: 1) reveal the hidden species diversity of the genus *Akanthomyces* according to phylogenetic analyses and morphological observation and 2) systematically review the geographical distribution and major host/substrate associations of *Akanthomyces* species by surveying the literature to the greatest extent possible and combining the results with those generated in our study.

## ﻿Materials and methods

### ﻿Soil and specimen collection

All of the soil samples were collected from Yunnan Province in China. Fungal specimens were obtained from six locations between 2017 and 2022, namely, two different locations in Yunnan Province, China, one location in Hunan Province, China, one location in Chiang Mai Province, Thailand, one location in Nghe An Province, Vietnam and one location in Oudomxay Province, Laos. Soil samples and specimens were noted and photographed in the field and then they were carefully put in plastic containers at a low temperature. After that, they were brought to the laboratory and stored at 4 °C prior to examination and isolation.

### ﻿Fungal isolation and culture

The *Akanthomyces* strains were isolated from the soil samples, based on the methods described by Wang et al. (2015) and Wang et al. (2023b). Briefly, 2 g of soil were added to a flask containing 20 ml sterilised water and glass beads. The soil suspension was shaken for about 10 min and then diluted 100 times. Subsequently, 200 µl of the diluted soil suspension was spread on Petri dishes with solidified onion garlic agar (OGA: 20 g of grated garlic and 20 g of onion were boiled in 1 litre of distilled water for 1 h; the boiled biomass was then filtered-off and 2% agar was added). Czapek yeast extract agar (CYA, Advanced Technology and Industrial Co., Ltd., China) and potato dextrose agar (PDA, Difco, USA) were used and all media had 50 mg/l rose Bengal and 100 mg/l kanamycin added. Conidia developing on invertebrate cadavers were transplanted on to plates of PDA and cultured at 25 °C. Colonies of the isolated filamentous fungi appearing in the culture were transferred on to fresh PDA media. Each purified fungal strain was transferred to PDA slants and cultured at 25 °C until its hyphae spread across the entire slope. The emerging fungal spores were washed with sterile physiological saline to form a suspension containing 1 × 10^3^ cells/ml. To obtain monospore cultures, a sample of the spore suspension was placed on PDA on a Petri dish utilising a sterile micropipette and then the dish was incubated at 25 °C. Voucher specimens and the corresponding isolated strains were deposited in the Yunnan Herbal Herbarium (**YHH**) and the Yunnan Fungal Culture Collection (YFCC), respectively, of Yunnan University, Kunming, China.

### ﻿Morphological observations

The specimens were examined with an Olympus SZ61 stereomicroscope (Olympus Corporation, Tokyo, Japan). Fungal structures of the specimens, such as synnemata, phialides and conidia, were mounted on glass slides with a drop of lactophenol cotton blue solution. Cultures on PDA slants were transferred to PDA plates and then they were incubated at 25 °C for 14 d. For morphological evaluation, microscope slides were prepared by placing mycelia from the cultures on PDA medium blocks (5 mm diameter) and then overlaid with a coverslip. Micro-morphological observations and measurements were performed with a light microscope (CX40, Olympus Corporation, Tokyo, Japan) and a scanning electron microscope (Quanta 200 FEG, FEI Company, Hillsboro, USA). The individual length and width measurements were recorded for 30–100 replicates and included the absolute minima and maxima.

### ﻿DNA extraction, PCR and sequencing

The specimens and axenic living cultures were prepared for DNA extraction. Genomic DNA was extracted utilising a Genomic DNA Purification kit (Qiagen GmbH, Hilden, Germany), based on the manufacturer’s instructions. The primer pair ITS5/ITS4 was used to amplify a fraction of the internal transcribed spacer regions of the rDNA (ITS rDNA) (White et al. 1990). Primer pair LR5/LR0R (Vilgalys and Hester 1990; Rehner and Samuels 1994) was used to amplify a fraction of the nuclear ribosomal large subunit (nrLSU) and EF1-983F/EF1-2218R primers (Rehner and Buckley 2005) were used to amplify translation elongation factor 1α (*TEF*). For amplification of the largest and second largest subunits of RNA polymerase ІІ (*RPB1* and *RPB2*), PCR primer pairs RPB1-5’F/RPB1-5’R and RPB2-5’F/RPB2-5’R (Bischoff et al. 2006; Sung et al. 2007) were employed. All of the PCR reactions were performed in a final volume of 50 μl and contained 25 μl of 2 × Taq PCR Master Mix (Tiangen Biotech Co., Ltd., Beijing, China), 0.5 μl of each primer (10 μM), 1 μl of genomic DNA and 23 μl of RNase-free water. Target gene amplification and sequencing were performed, based on the methods detailed in our prior study (Wang et al. 2020).

### ﻿Phylogenetic analyses

The phylogenetic analyses were based on five genes, namely, ITS, nrLSU, *TEF*, *RPB1* and *RPB2*, sequences. The sequences were retrieved from GenBank (http://www.ncbi.nlm.nih.gov/, accessed on 1 March 2023) and combined with those generated in our study. Taxon information and GenBank accession numbers are listed in Table 1. Sequences were aligned with MAFFT v.7 (http://mafft.cbrc.jp/alignment/server/, accessed on 1 March 2023). The aligned sequences were then manually corrected when necessary. After alignment, the sequences of the genes were concatenated. Conflicts amongst the five genes were resolved with PAUP* 4.0b10 (Swofford et al. 2002). The results showed that the phylogenetic signals for the five loci were congruent (*P* = 0.02). The data partitions were defined for the combined dataset with PartitionFinder v.1.1.1 (Lanfear et al. 2012). Phylogenetic analyses were conducted utilising Bayesian Inference (BI) and Maximum Likelihood (ML) methods, respectively. The model selected for BI analysis was from jModelTest version 2.1.4 (Darriba et al. 2012). The following models were implemented in the analysis: GTR + I + G for partitions of ITS, nrLSU and *TEF* and GTR + I for partitions of *RPB1* and *RPB2*. The BI analysis was executed on MrBayes v.3.2.7a for five million generations (Ronquist et al. 2012). GTR + FO + G was selected as the optimal model for ML analysis and 1000 rapid bootstrap replicates were performed on the dataset. ML phylogenetic analyses were conducted in RAxML 7.0.3 (Stamatakis et al. 2008). Additional ML analyses were performed using IQ-TREE v. 2.1.3 with ultrafast bootstrapping for the estimation of branch support (Minh et al. 2020). Further, ML analysis (IQ-TREE) was applied to single-locus genealogies for ITS, nrLSU, *TEF*, *RPB1* and *RPB2*.

**Table 1.** Specimen information and GenBank accession numbers for sequences used in this study.

Species	Voucher information	Host/Substrate	GenBank accession numbers					Reference
			ITS	nrLSU	*TEF*	*RPB1*	*RPB2*	
*Akanthomycesaculeatus*	HUA 186145	–	–	MF416520	MF416465	–	–	Kepler et al. (2017)
*Akanthomycesaculeatus*	TS772	Lepidoptera; Sphingidae	KC519371	KC519370	KC519366	–	–	Sanjuan et al. (2014)
*Akanthomycesaraneicola*	GY29011^T^	Araneae; spider	MK942431	–	MK955950	MK955944	MK955947	Chen et al. (2019)
*Akanthomycesaraneogenus*	GZUIF DX2^T^	Araneae; spider	MH978179	–	MH978187	MH978182	MH978185	Chen et al. (2018)
***Akanthomycesaraneogenus***	**YFCC 1811934**	**Araneae; spider**	**OQ509518**	**OQ509505**	**OQ506281**	**OQ511530**	**OQ511544**	**This study**
	**YFCC 2206935**	**Araneae; spider**	**OQ509519**	**OQ509506**	**OQ506282**	**OQ511531**	**OQ511545**	**This study**
*Akanthomycesaraneosus*	KY11341^T^	Araneae; spider	ON502826	ON502832	ON525443	–	ON525442	Chen et al. (2022)
*Akanthomycesattenuatus*	CBS 170.76^T^	Lepidoptera; *Carpocapsapomonella*	MH860970	OP752153	OP762607	OP762611	OP762615	Manfrino et al. (2022)
*Akanthomycesbashanensis*	CQ05621^T^	Araneae; spider	OQ300412	OQ300420	OQ325024	–	OQ349684	Chen et al. (2023)
*Akanthomycesbeibeiensis*	CQ05921^T^	Araneae; spider	OQ300415	OQ300424	OQ325028	–	OQ349688	Chen et al. (2023)
*Akanthomycescoccidioperitheciatus*	NHJ 6709	Araneae; spider	JN049865	EU369042	EU369025	EU369067	EU369086	Kepler et al. (2012)
*Akanthomycesdipterigenus*	CBS 126.27	Hemiptera; *Iceryapurchasi*	AJ292385	KM283797	KM283820	KR064300	KM283862	Kepler et al. (2017)
***Akanthomycesdipterigenus***	**YFCC 2107933**	**Soil**	**OQ509520**	**OQ509507**	**OQ506283**	**OQ511532**	**OQ511546**	**This study**
*Akanthomyceskanyawimiae*	TBRC 7242	Araneae; spider	MF140751	MF140718	MF140838	MF140784	MF140808	Mongkolsamrit et al. (2018)
	TBRC 7243	Unidentified	MF140750	MF140717	MF140837	MF140783	MF140807	Mongkolsamrit et al. (2018)
***Akanthomyceskunmingensis***	**YFCC 1708939**	**Araneae; spider**	**OQ509521**	**OQ509508**	**OQ506284**	**OQ511533**	**OQ511547**	**This study**
	**YFCC 1808940^T^**	**Araneae; spider**	**OQ509522**	**OQ509509**	**OQ506285**	**OQ511534**	**OQ511548**	**This study**
***Akanthomyceslaosensis***	**YFCC 1910941^T^**	**Lepidoptera; Noctuidae**	**OQ509523**	**OQ509510**	**OQ506286**	**OQ511535**	**OQ511549**	**This study**
	**YFCC 1910942**	**Lepidoptera; Noctuidae**	**OQ509524**	**OQ509511**	**OQ506287**	**OQ511536**	**OQ511550**	**This study**
*Akanthomyceslecanii*	CBS 101247	Hemiptera; *Coccusviridis*	JN049836	AF339555	DQ522359	DQ522407	DQ522466	Kepler et al. (2012)
*Akanthomyceslepidopterorum*	GZAC SD05151^T^	Lepidoptera (pupa)	MT705973	–	–	–	MT727044	Chen et al. (2020b)
*Akanthomycesmuscarius*	CBS 455.70B	–	–	MH871560	–	–	–	Kepler et al. (2017)
*Akanthomycesneoaraneogenus*	GZU1031Lea^T^	Araneae; spider	KX845703	–	KX845697	KX845699	KX845701	Chen et al. (2017)
*Akanthomycesneocoleopterorum*	GY11241^T^	Coleoptera	MN093296	–	MN097813	MN097816	MN097812	Chen et al. (2020a)
	GY11242	Coleoptera	MN093298	–	MN097815	MN097817	MN097814	Chen et al. (2020a)
*Akanthomycesnoctuidarum*	BCC 36265^T^	Lepidoptera; Noctuidae	MT356072	MT356084	MT477978	MT477994	MT477987	Aini et al. (2020)
	BCC 47498	Lepidoptera; Noctuidae	MT356074	MT356086	MT477980	MT477996	MT477988	Aini et al. (2020)
	BCC 28571	Lepidoptera; Noctuidae	MT356075	MT356087	MT477981	MT478009	MT478006	Aini et al. (2020)
*Akanthomycespissodis*	CBS 118231^T^	Coleoptera; *Pissodesstrobi*	–	KM283799	KM283822	KM283842	KM283864	Chen et al. (2020b)
***Akanthomycespseudonoctuidarum***	**YFCC 1808943^T^**	**Lepidoptera; Noctuidae**	**OQ509525**	**OQ509512**	**OQ506288**	**OQ511537**	**OQ511551**	**This study**
	**YFCC 1808944**	**Lepidoptera; Noctuidae**	**OQ509526**	**OQ509513**	**OQ506289**	**OQ511538**	**OQ511552**	**This study**
*Akanthomycespyralidarum*	BCC 28816^T^	Lepidoptera; Pyralidae	MT356080	MT356091	MT477982	MT478000	MT478007	Aini et al. (2020)
	BCC 32191	Lepidoptera; Pyralidae	MT356081	MT356092	MT477983	MT478001	MT477989	Aini et al. (2020)
*Akanthomycessabanensis*	ANDES-F 1023	Hemiptera; *Pulvinariacaballeroramosae*	KC633237	–	KC633267	KC875222	–	Kepler et al. (2017)
	ANDES-F 1024	Hemiptera; *Pulvinariacaballeroramosae*	KC633232	KC875225	KC633266	–	KC633249	Kepler et al. (2017)
***Akanthomyces* sp.**	**YFCC 945**	**Soil**	**OQ509531**	–	**OQ506294**	**OQ511543**	**OQ511557**	**This study**
***Akanthomycessubaraneicola***	**YFCC 2107937^T^**	**Araneae; spider**	**OQ509527**	**OQ509514**	**OQ506290**	**OQ511539**	**OQ511553**	**This study**
	**YFCC 2107938**	**Araneae; spider**	**OQ509528**	**OQ509515**	**OQ506291**	**OQ511540**	**OQ511554**	**This study**
*Akanthomycessulphureus*	TBRC 7248^T^	Araneae; spider	MF140758	MF140722	MF140843	MF140787	MF140812	Mongkolsamrit et al. (2018)
	TBRC 7249	Araneae; spider	MF140757	MF140721	MF140842	MF140786	MF140734	Mongkolsamrit et al. (2018)
***Akanthomycessulphureus***	**YFCC 1710936**	**Araneae; spider**	**OQ509529**	**OQ509516**	**OQ506292**	**OQ511541**	**OQ511555**	**This study**
*Akanthomycesthailandicus*	TBRC 7245^T^	Araneae; spider	MF140754	–	MF140839	–	MF140809	Mongkolsamrit et al. (2018)
*Akanthomycestiankengensis*	KY11571^T^	Araneae; spider	ON502848	ON502825	ON525447	–	ON525446	Chen et al. (2022)
	KY11572	Araneae; spider	ON502821	ON502827	ON525449	–	ON525448	Chen et al. (2022)
*Akanthomycestortricidarum*	BCC 72638^T^	Lepidoptera; Tortricidae	MT356076	MT356088	MT478004	MT477997	MT477992	Aini et al. (2020)
	BCC 41868	Lepidoptera; Tortricidae	MT356077	MT356089	MT477985	MT477998	MT478008	Aini et al. (2020)
*Akanthomycestuberculatus*	HUA 186131	Lepidoptera (adult moth)	–	MF416521	MF416466	–	–	Kepler et al. (2017)
*Akanthomycesuredinophilus*	KACC 44066	Rust	–	KM283784	KM283808	KM283830	KM283850	Park et al. (2016)
	KACC 44082^T^	Rust	–	KM283782	KM283806	KM283828	KM283848	Park et al. (2016)
	KUN 101466	Insect	MG948305	MG948307	MG948315	MG948311	MG948313	Park et al. (2016)
	KUN 101469	Insect	MG948306	MG948308	MG948316	MG948312	MG948314	Park et al. (2016)
*Akanthomyceswaltergamsii*	TBRC 7251	Araneae; spider	MF140747	MF140713	MF140833	MF140781	MF140805	Mongkolsamrit et al. (2018)
	TBRC 7252^T^	Araneae; spider	MF140748	MF140714	MF140834	MF140782	MF140806	Mongkolsamrit et al. (2018)
***Akanthomyceswaltergamsii***	**YFCC 883**	**Araneae; spider**	**OQ509530**	**OQ509517**	**OQ506293**	**OQ511542**	**OQ511556**	**This study**
*Akanthomyceszaquensis*	HMAS 246915^T^	Fungi; *Ophiocordycepssinensis*	MT789699	MT789697	MT797812	MT797810	–	Wang et al. (2023a)
	HMAS 246917	Fungi; *Ophiocordycepssinensis*	MT789698	MT789696	MT797811	MT797809	–	Wang et al. (2023a)
*Samsoniellaaurantia*	TBRC 7271^T^	Lepidoptera	MF140764	MF140728	MF140846	MF140791	MF140818	Mongkolsamrit et al. (2018)
*Samsoniellainthanonensis*	TBRC 7915^T^	Lepidoptera (pupa)	MF140761	MF140725	MF140849	MF140790	MF140815	Mongkolsamrit et al. (2018)

Boldface: data generated in this study. Ex-type materials are marked with “T”.

### ﻿Identification of host arthropods

The host arthropods of *Akanthomyces* spp. were identified on the basis of morphological characteristics and they were further identified utilising molecular analyses according to the mitochondrial cytochrome oxidase I gene (*cox1*) and mitochondrial cytochrome b gene (*cytb*). Genomic DNA was extracted from the head and leg areas of the cadavers of the hosts by utilising the CTAB method (Liu et al. 2001). The *cox1* and *cytb* loci were amplified with the primer pair Hep-cox1F/Hep-cox1R and Hep-cytbF/Hep-cytbR, respectively (Simon et al. 1994). Sequences were analysed with MEGA v.6.06 software (Tamura et al. 2013) and processed by Standard Nucleotide BLAST (GenBank, NCBI nucleotide database) to assess similarity with reported arthropod sequences.

## ﻿Results

### ﻿Sequencing and phylogenetic analyses

**Figure 1.**
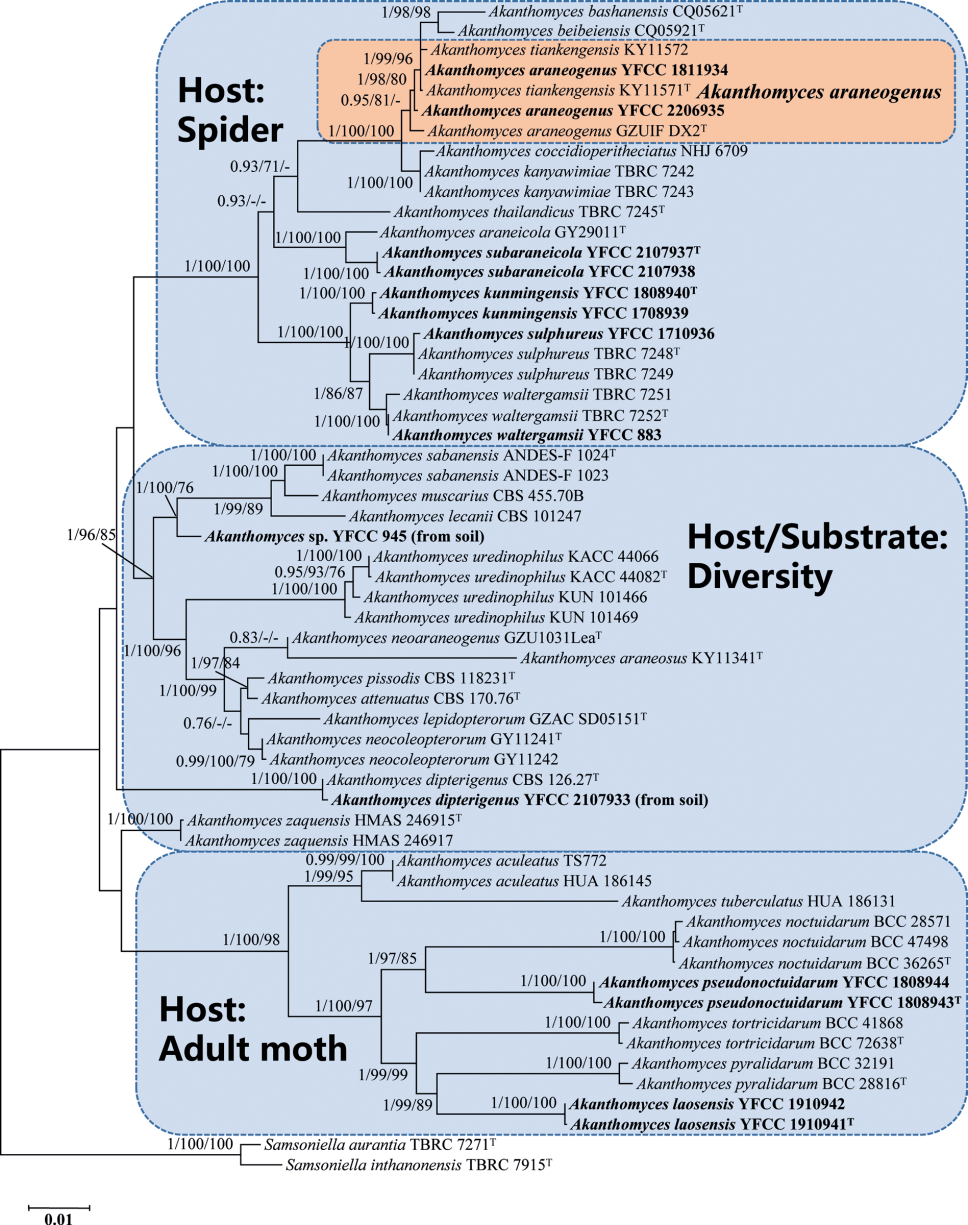
Phylogenetic tree of *Akanthomyces* species, based on combined partial ITS + nrLSU + *TEF* + *RPB1* + *RPB2* sequences. Numbers at the branches indicate support values (BI-PP/IQ-TREE-BS/ RAxML-BS) above 0.7/70%/70%. Ex-type materials are marked with “T”. Isolates in bold type are those analysed in this study.

The five DNA loci (ITS, nrLSU, *TEF*, *RPB1*, *RPB2*) were readily amplified and sequenced and there was a fairly high success rate in this study. Preliminary phylogenetic analyses, based on the combined five-gene sequences from 116 fungal taxa Cordycipitaceae and *Trichoderma* Pers., confirmed the presence and positions of *Akanthomyces* and related genera within Cordycipitaceae. The concatenated five-gene dataset consisted of 4,453 bp (ITS = 639 bp, nrLSU = 921 bp, *TEF* = 1,044 bp, *RPB1* = 758 bp and *RPB2* = 1,091 bp). Ten well-supported clades were recognized, which accommodate species of the genera *Akanthomyces*, *Ascopolyporus* Möller, *Beauveria* Vuill., *Blackwellomyces* Spatafora & Luangsa-ard, *Cordyceps*, *Gibellula* Cavara, *Hevansia*, *Samsoniella*, *Simplicillium* W. Gams & Zare and *Trichoderma* (Suppl. material 1: fig. S1). The phylogenetic analyses also revealed the species diversity of the genus *Akanthomyces*. This suggested that the group should be genetically composed of at least 30 species (Suppl. material 1: fig. S1). The further phylogenetic analyses, based on combined partial ITS+nrLSU+*TEF*+*RPB1*+*RPB2* sequences consisting of 56 fungal taxa (Table 1), resolved the majority of the *Akanthomyces* lineages into separate terminal branches (Fig. 1). The dataset consisted of 4,401 bp of sequence data (ITS = 619 bp, nrLSU = 896 bp, *TEF* = 1,022 bp, *RPB1* = 731 bp and *RPB2* = 1,133 bp). *Samsoniellaaurantia* Mongkols., Noisrip., Thanakitp., Spatafora & Luangsa-ard (strain TBRC 7271) and *S.inthanonensis* Mongkols., Noisrip., Thanakitp., Spatafora & Luangsa-ard (strain TBRC 7915) within Cordycipitaceae were used as the outgroup sequences for this dataset. This revealed a similar tree and cluster topology, as shown in Suppl. material 1: fig. S1. Amongst the hosts of *Akanthomyces*, Araneae (spider) and Lepidoptera (adult moth) are the two major orders. Most of the spider pathogens form a monophyletic clade, separated from the pathogens of moths, themselves forming also an apparent monophyletic clade (Fig. 1). The phylogenetic analyses also suggested the existence of distinct species in the spider pathogens and adult moth entomopathogens clade that we proposed as new species: *A.kunmingensis* and *A.subaraneicola*, which were found in the spider pathogens clade; and *A.laosensis* and *A.pseudonoctuidarum*, which were found in the adult moth entomopathogens clade (Fig. 1).

Despite differing topologies between individual loci (ITS, nrLSU, *TEF*, *RPB1* and *RPB2*), the newly-proposed species usually stood out as distinct clades to other known species. Some novel species always recovered the sister relationship to a particular known species for all loci. For example, the newly-discovered species *A.kunmingensis* had a close genetic relationship with *A.waltergamsii*. They were regarded as different species with strong support from ITS, nrLSU, *TEF*, *RPB1* and *RPB2* (Suppl. material 1: figs S2–S6). The new species *A.subaraneicola* was sisters to *A.araneicola* and this relationship received significant bootstrap support from ITS, *TEF*, *RPB1* and *RPB2* (Suppl. material 1: figs S2, S4–S6). Meanwhile, *A.laosensis* was inferred to form a sister clade to either *A.pyralidarum* (ITS, *RPB1* and *RPB2*) or *A.tortricidarum* (nrLSU and *TEF*). Similarly, despite the differing position of *A.pseudonoctuidarum* between different markers, it always formed a clade that could be distinguished from its closely-related species, *A.noctuidarum* and *A.tortricidarum*.

### ﻿Morphological features

**Figure 2.**
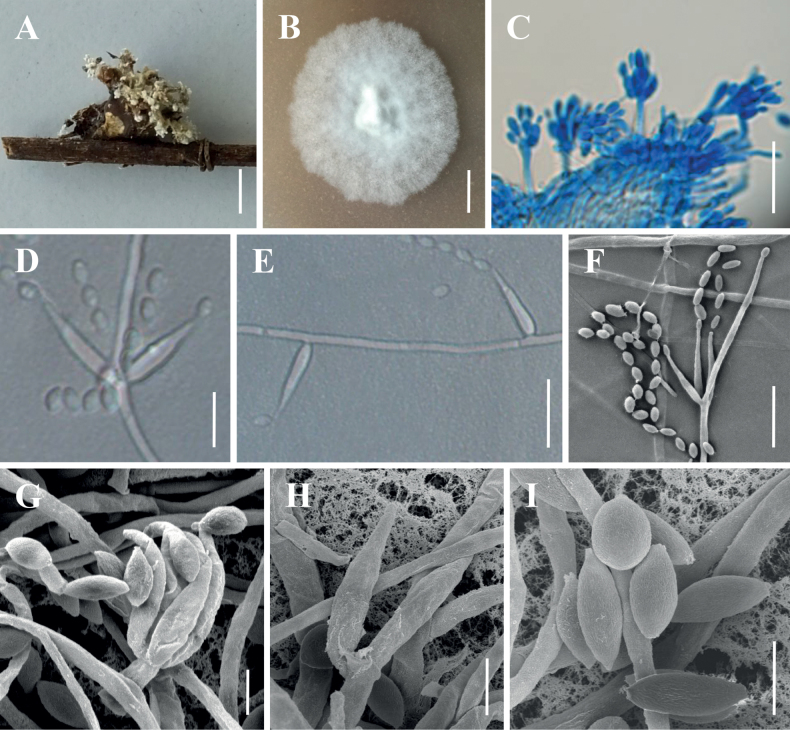
Morphology of *Akanthomyceskunmingensis***A** the type specimen (YHH 16988) **B** culture character on PDA medium **C** conidiogenous structures on the host **D–H** conidiophores, conidiogenous cells and conidia **I** conidia. Scale bars: 3 mm (**A**); 10 mm (**B**); 10 µm (**C, E, F**); 5 µm (**D**); 2 µm (**G–I**).

**Figure 3.**
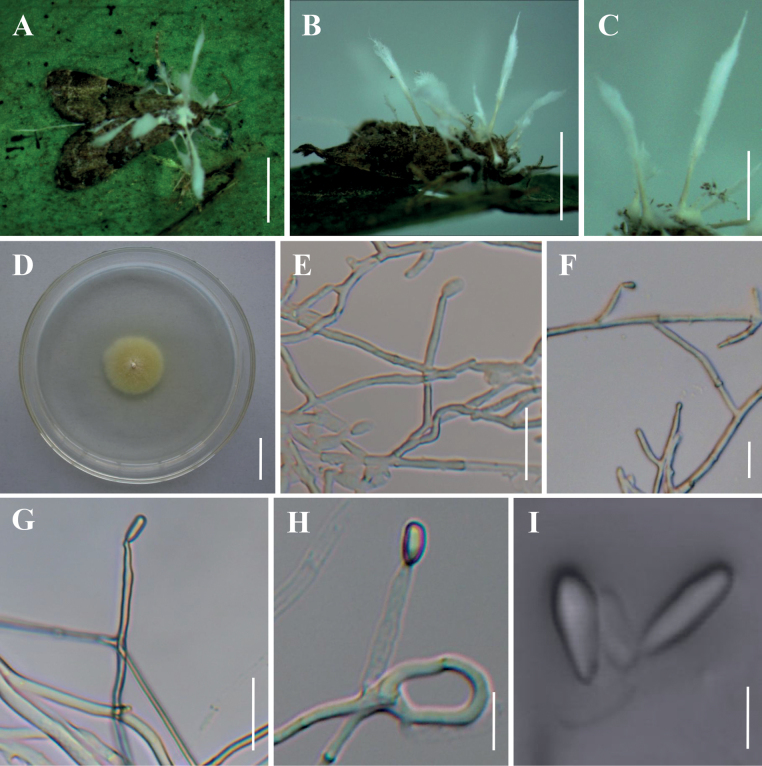
Morphology of *Akanthomyceslaosensis***A, B** fungus on adult moth **C** long synnemata **D** culture character on PDA medium **E–H** conidiophores, conidiogenous cells and conidia **I** conidia from long synnemata. Scale bars: 10 mm (**A, B**); 5 mm (**C**); 20 mm (**D**); 20 µm (**E–G**); 10 µm (**H**); 5 µm (**I**).

**Figure 4.**
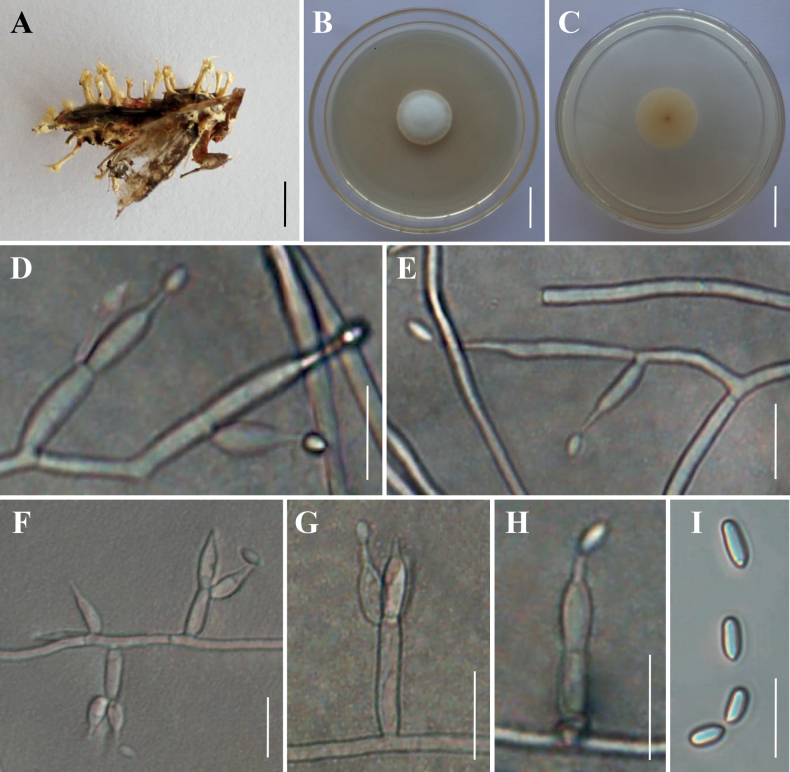
Morphology of *Akanthomycespseudonoctuidarum***A** adult moth infected by *A.pseudonoctuidarum***B, C** culture character on PDA medium **D–H** conidiophores, conidiogenous cells and conidia **I** conidia. Scale bars: 2 mm (**A**); 20 mm (**B, C**); 10 µm (**D–I**).

**Figure 5.**
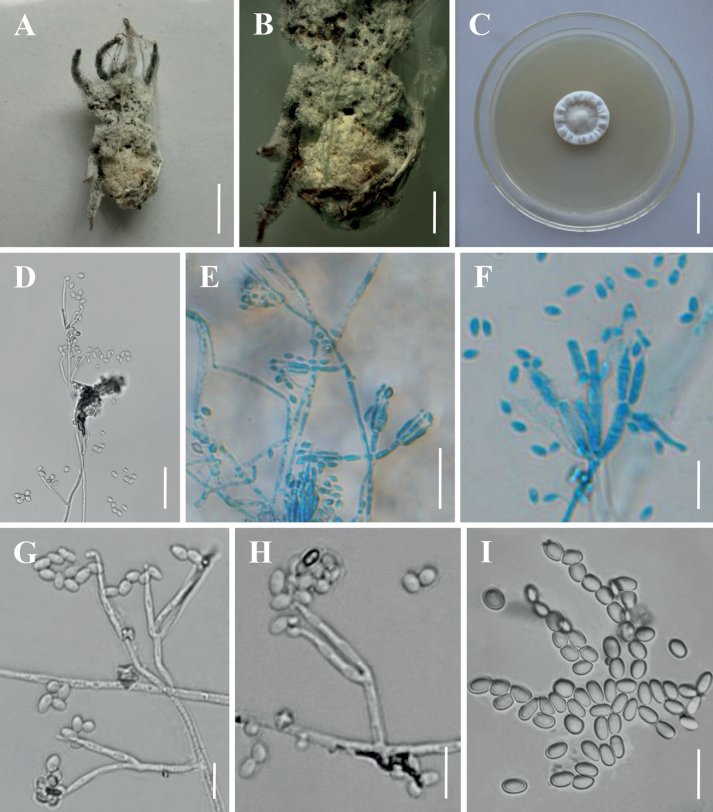
Morphology of *Akanthomycessubaraneicola***A, B** fungus on spider **C** culture character on PDA medium **D–H** conidiophores, conidiogenous cells and conidia **I** conidia. Scale bars: 10 mm (**A**); 5 mm (**B**); 20 mm (**C**); 30 µm (**D**); 20 µm (**E**); 10 µm (**F–I**).

**Figure 6.**
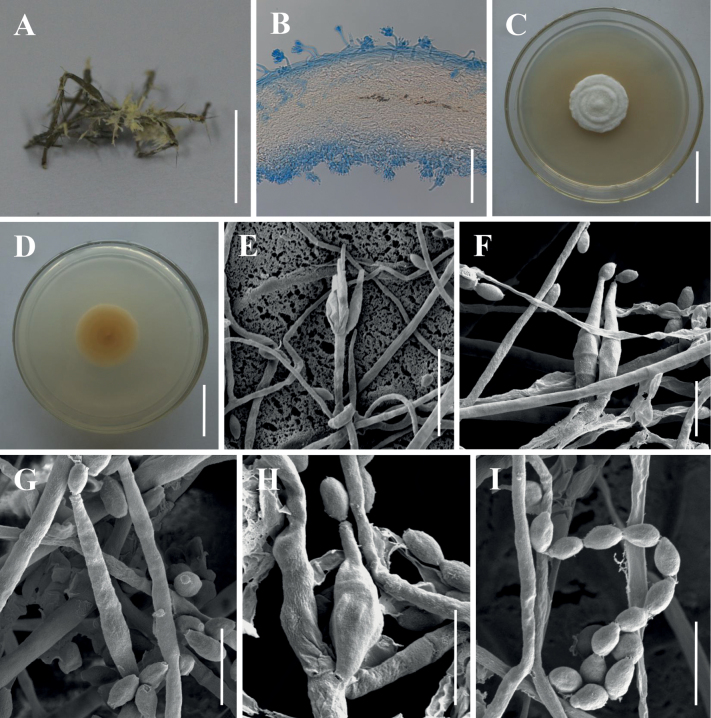
Morphology of *Akanthomycesaraneogenus***A** fungus on spider **B** conidiogenous structures on the host **C,D** culture character on PDA medium **E–H** conidiophores, conidiogenous cells and conidia **I** conidia. Scale bars: 5 mm (**A**); 30 µm (**B**); 30 mm (**C, D**); 10 µm (**E**); 5 µm (**F–I**).

The morphological characteristics of the five species, as well as photomicrographs of morphological structures, are shown in Figs 2–6. The detailed fungal morphological descriptions are supplied in the Taxonomy section.

## ﻿Taxonomy

### ﻿ Akanthomyces kunmingensis

Taxon classificationFungiHypocrealesCordycipitaceae

Hong Yu bis, Y. Wang & Z.Q. Wang sp. nov.

F592BD4C-1006-57D0-81E9-739478AF39A3

848307

Fig. 2

#### Etymology.

Named after the location, Kunming City, where the species was collected.

#### Type.

China. Yunnan Province, Kunming City, Wild Duck Lake Forest Park (25.2181°N, 102.8503°E, 2100 m above sea level), on a spider on a dead stem, 14 August 2018, collected by Yao Wang (holotype: YHH 16988; ex-type living culture: YFCC 1808940).

#### Description.

***Sexual morph***: Undetermined. ***Asexual morph***: Synnemata arising from spider body, cream to light yellow, erect, irregularly branched, producing a mass of conidia at the upper apex, powdery and floccose. Colonies on PDA reaching 15–20 mm in diameter after 14 days at 25 °C, circular, white and fluffy mycelium, middle bulge, reverse pale yellow to light brown. Hyphae smooth‐walled, branched, septate, hyaline, 0.5–2.8 μm wide. Conidiophores smooth‐walled, cylindrical, solitary, sometimes verticillate, 4.3–9.5 × 1.2–2.0 μm (n = 30). Phialides consisting of a cylindrical, somewhat inflated base, verticillate on conidiophores, usually in whorls of 4–5 or solitary on hyphae, 6.2–29.4 × 1.1–2.5 μm (n = 30). Conidia smooth and hyaline, ellipsoidal to long oval, one‐celled, 1.9–3.5 × 1.1–1.8 μm (n = 50), often in chains. Size and shape of phialides and conidia similar in culture and on natural substratum.

#### Host.

Spider (Araneae).

#### Habit.

On spiders on dead stems.

#### Distribution.

Kunming City, Yunnan Province, China.

#### Other material examined.

China. Yunnan Province, Kunming City, Songming County, Dashao Village (25.3924°N, 102.5589°E, 2700 m above sea level), on a spider on a dead stem, 12 August 2017, Yao Wang (YHH 2301006; living culture: YFCC 1708939).

#### Commentary.

In regard to phylogenetic relationships, *Akanthomyceskunmingensis* forms a distinct lineage in the genus *Akanthomyces* with high credible support (1/100%/100%) and it is closely related to *A.sulphureus* and *A.waltergamsii* (Fig. 1). Morphologically, *A.kunmingensis* is so similar to *A.waltergamsii* that it was once referred to as *A.waltergamsii* by Wang et al. (2020); however, a morphological observation revealed a significant difference of conidia shapes between *A.kunmingensis* and *A.waltergamsii*. *Akanthomyceskunmingensis* usually produces a variety of shapes of conidia (viz. spherical, ellipsoidal to long oval or fusiform), while *A.waltergamsii* produces only ellipsoidal and fusiform conidia. Moreover, *A.kunmingensis* can be distinguished from *A.sulphureus* and *A.waltergamsii* by its longer phialides (6.2–29.4 µm) and smaller conidia (1.9–3.5 × 1.1–1.8 μm) (Table 3).

### ﻿ Akanthomyces laosensis

Taxon classificationFungiHypocrealesCordycipitaceae

Hong Yu bis & Y. Wang sp. nov.

FDFDC04F-B2E7-5A3E-B58B-467C4921CB98

848308

Fig. 3

#### Etymology.

Named after the location, Laos, where the species was collected.

#### Type.

Laos. Oudomxay Province, Muang Xay County, Nagang Village (20.7143°N, 102.0957°E, 698 m above sea level), on the adult of Noctuidae on the underside of a dicotyledonous leaf, 5 October 2019, collected by Yao Wang (holotype: YHH 2301008; ex-holotype living culture: YFCC 1910941).

#### Description.

***Sexual morph***: Undetermined. ***Asexual morph***: Specimens examined in this study can be found on the underside of dicotyledonous leaves. Synnemata arose at the head and in the middle of the host body, white, up to 15.6 mm long and 0.6–1.3 mm wide, rarely branched, feathery to clavate with acute or blunt ends. Colonies on PDA moderately fast-growing at 25 °C, reaching 23–26 mm in diameter in 14 days, circular, flat, white in the middle with a light yellow edge, reverse light yellow. Hyphae smooth-walled, branched, septate, hyaline, 0.8–3.5 µm wide. Conidiogenous cells monophialidic, produced along the synnemata or solitary on hyphae in culture. Phialides smooth-walled, hyaline, cylindrical, 11.5–30.0 × 2.0–4.2 µm (n = 30). Conidia smooth and hyaline, cylindrical or long oval, one-celled, 4.1–9.8 × 2.3–4.2 µm (n = 30). Size and shape of phialides and conidia similar in culture and on natural substratum.

#### Host.

Adult moth (Noctuidae, Lepidoptera).

#### Habit.

On the adults of Noctuidae sp. on the underside of leaves of plants.

#### Distribution.

Muang Xay County, Oudomxay Province, Laos.

#### Other material examined.

Laos. Oudomxay Province, Muang Xay County, Nam Kit Park (20.6651°N, 102.0007°E, 695 m above sea level), on an adult moth on the underside of a leaf, 1 October 2019, Yao Wang (YHH 2301000; living culture: YFCC 1910942).

#### Commentary.

Phylogenetically, *Akanthomyceslaosensis* forms a distinct lineage and is closely related to *A.pyralidarum* with strong statistical support (1/99%/89%) (Fig. 1). Morphologically, *A.laosensis* is distinctly different from *A.pyralidarum* because of its longer synnemata (up to 15.6 mm). Furthermore, *A.laosensis* was determined to occur on an adult of Noctuidae sp., while *A.pyralidarum* was located on an adult of Pyralidae sp. In fact, the species is easily distinguished from other known species in the genus of *Akanthomyces* by its longer phialides (11.5–30.0 µm) and larger conidia (4.1–9.8 × 2.3–4.2 µm) (Table 3).

### ﻿ Akanthomyces pseudonoctuidarum

Taxon classificationFungiHypocrealesCordycipitaceae

Hong Yu bis & Y. Wang sp. nov.

4C9B1F4B-7FB4-56F6-B1F6-06CBC5D0FDF9

848309

Fig. 4

#### Etymology.

Referring to macromorphological resemblance of *A.noctuidarum*, but *A.pseudonoctuidarum* is phylogenetically distinct.

#### Type.

Thailand. Chiang Mai Province, Chiang Mai City, Sansai District, Maejo Farm (18.9177°N, 99.0520°E, 317 m above sea level), on the adult of Noctuidae on the underside of a dicotyledonous leaf, 22 August 2018, collected by Hong Yu (holotype: YHH 2301010; ex-type living culture: YFCC 1808943).

#### Description.

***Sexual morph***: Undetermined. ***Asexual morph***: Synnemata arising from moth body, cream to light yellow, erect, simple, cylindrical to clavate, 800–2000 × 120–350 µm. Conidia and reproductive structures on natural substratum not observed. Colonies on PDA moderately fast-growing at 25 °C, reaching a diameter of 25–28 mm within 14 days, circular, flat to raised, white and fluffy mycelium, reverse cream to pale yellow. Hypha smooth-walled, hyaline, septate, 1.0–2.9 µm wide. Conidiophores smooth-walled, cylindrical, solitary, 6.5–13.8 × 1.8–3.6 µm (n = 30). Conidiogenous cells monophialidic or polyphialidic. Phialides verticillate, usually in whorls of 2–3 or solitary on hyphae, cylindrical with papillate end, hyaline, 6.8–26.0 × 2.1–3.6 µm (n = 30). Conidia smooth and hyaline, ellipsoidal to long oval, one-celled, 2.6–6.4 × 1.5–2.2 µm (n = 30).

#### Host.

Adult moth (Noctuidae, Lepidoptera).

#### Habit.

On the adults of Noctuidae sp. on the underside of leaves of plants.

#### Distribution.

Chiang Mai City, Chiang Mai Province, Thailand.

#### Other material examined.

Thailand, Chiang Mai Province, Chiang Mai City, Mae Rim District, Queen Sirikit Botanic Garden (18.8990°N, 98.8605°E, 536 m above sea level), on an adult of Noctuidae, 26 August 2018, collected by Yao Wang (YHH 2301011; living culture: YFCC 1808944).

#### Commentary.

*Akanthomycespseudonoctuidarum* is similar to its phylogenetically closely-related species *A.noctuidarum* in macromorphology. They have the same hosts (the adults of Noctuidae sp.) and *Isaria*-like asexual conidiogenous structures, producing cream or light yellow synnemata. However, *A.pseudonoctuidarum* is easily recognised by its larger synnemata (800–2000 × 120–350 µm), longer phialides (6.8–26.0 µm) and larger conidia (2.6–6.4 × 1.5–2.2 µm) (Table 3). It was easily distinguished phylogenetically from *A.noctuidarum* (Fig. 1; 1/97%/85%). Both the morphological study and phylogenetic analyses of combined ITS, nrLSU, *TEF*, *RPB1* and *RPB2* sequence data supported that this fungus is a distinct species in the genus *Akanthomyces*.

### ﻿ Akanthomyces subaraneicola

Taxon classificationFungiHypocrealesCordycipitaceae

Hong Yu bis, Y. Wang & Z.Q. Wang sp. nov.

D96142BB-5D7A-5A32-B454-F03B6B082D65

848310

Fig. 5

#### Etymology.

“Subaraneicola” refers to morphologically resembling *A.araneicola*, but phylogenetically distinct.

#### Type.

China. Hunan Province, Huaihua City, Zhongpo National Forest Park (27.5724°N, 109.9664°E, 615 m above sea level), on a spider emerging from leaf litter on the forest floor, 10 July 2021, collected by Yao Wang (holotype: YHH 2301004; ex-type living culture: YFCC 2107937).

#### Description.

***Sexual morph***: Undetermined. ***Asexual morph***: Mycosed hosts covered by white to pale yellow mycelia, producing numerous powdery conidia, synnemata not observed. Colonies on PDA reaching 24–28 mm in diameter within 14 days at 25 °C, circular, white and fluffy mycelium in the centre, cottony with a raised mycelial density at the outer ring, reverse white to pale yellow. Hyphae smooth‐walled, branched, septate, hyaline, 1.6–3.2 μm wide. Conidiophores smooth‐walled, cylindrical, solitary, sometimes verticillate, 6.5–12.3 × 1.6–3.5 μm (n = 30). Conidiogenous cells monophialidic or polyphialidic. Phialides consisting of a cylindrical, somewhat inflated base, verticillate on conidiophores, usually in whorls of 2–5, or solitary on hyphae, 12.1–38.2 × 1.3–3.2 μm (n = 30). Conidia smooth and hyaline, ellipsoidal to long oval, one‐celled, 3.0–5.4 × 1.8–3.4 μm (n = 50), often in chains. Size and shape of phialides and conidia similar in culture and on natural substratum.

#### Host.

Spider (Araneae).

#### Habit.

On spiders on dead stems or emerging from leaf litter on the forest floor.

#### Distribution.

Hunan and Yunnan Province, China.

#### Other material examined.

China, Yunnan Province, Kunming City, Wild Duck Lake Forest Park (25.1244°N, 102.8716°E, 1900 m above sea level), on a spider on a dead stem, 28 July 2021, Yao Wang (YHH 2301005; living culture: YFCC 2107938).

#### Commentary.

Morphologically, *Akanthomycessubaraneicola* resembles the phylogenetic sister species *A.araneicola*. They were found to be parasitic on spiders (Araneae) and they are easily recognised by having white to pale yellow mycelia covering the hosts with a mass of conidia; however, our morphological observation revealed a significant difference in the shape and size of conidia between *A.subaraneicola* and *A.araneicola*. *Akanthomycessubaraneicola* usually produces large ellipsoidal to long oval conidia (3.0–5.4 × 1.8–3.4 μm), while *A.araneicola* produces small fusiform conidia (2.5–5.0 × 1.3–1.9 μm) (Table 3). In addition, molecular phylogenetic analyses indicated that they are distinct species (Fig. 1; 1/100%/100%).

### ﻿ Akanthomyces araneogenus

Taxon classificationFungiHypocrealesCordycipitaceae

Z.Q. Liang, W.H. Chen & Y.F. Han, Phytotaxa 379(1): 69 (2018)

BAFFDD6C-E05A-5C92-90DF-A47A8E8DE386

816114

Fig. 6

Akanthomyces tiankengensis W.H. Chen, Y.F. Han, J.D. Liang & Z.Q. Liang, Microbiology Spectrum 10(5): e01975-22, 6 (2022). Synonym.

#### Description.

***Sexual morph***: Undetermined. ***Asexual morph***: Mycosed hosts covered with white to pale yellow mycelia, occasionally several synnemata arising from all of the parts of the host. Colonies on PDA moderately fast-growing at 25 °C, reaching a diameter of 25–36 mm in 14 days at 25 °C, circular, middle bulge, white to yellowish, reverse yellowish. Hyphae smooth‐walled, branched, septate, hyaline, 0.5–2.9 μm wide. Conidiophores smooth‐walled, cylindrical, solitary, 10.6–22.4 × 1.3–2.6 μm (n = 30). Phialides consisting of a cylindrical, somewhat inflated base, verticillate on conidiophores, usually in whorls of 2–3 or solitary on hyphae, 8.1–17.8 × 1.1–3.6 μm (n = 30). Conidia smooth and hyaline, one‐celled, globose, 1.6–2.4 μm in diameter or ellipsoidal to fusiform, 2.2–4.1 × 1.1–2.3 μm (n = 50), often in chains. Size and shape of phialides and conidia similar in culture and on natural substratum.

#### Host.

Spider (Araneae).

#### Habit.

On the spiders on dead stems or emerging from leaf litter.

#### Distribution.

Guizhou and Yunnan Province, China; Chiang Mai Province, Thailand; Nghe An Province, Vietnam.

#### Material examined.

Thailand, Chiang Mai Province, Chiang Mai City, Queen Sirikit Botanic Garden (18.8990°N, 98.8604°E, 547 m above sea level), on a spider on a dead stem, 20 November 2018, Yao Wang (YHH 2301001; living culture: YFCC 1811934). VIETNAM, Nghe An Province, Pu Mat National Park (18.9292°N, 104.5889°E, 621 m above sea level), on spiders emerging from leaf litter on the forest floor, 28 April 2017, Yao Wang (YHH 2301007, YHH 2301012; living culture: YFCC 1704946, YFCC 1704947). China, Yunnan Province, Dai Autonomous Prefecture of Xishuangbanna, Mengla County (21.1817°N, 101.7252°E, 875 m above sea level), on a spider on a dead stem, 12 June 2022, Zhi-Qin Wang (YHH 2301002; living culture: YFCC 2206935).

#### Commentary.

In our phylogenetic analyses, *Akanthomycesaraneogenus* ex-type strain (GZUIF DX2) and *A.tiankengensis* ex-type isolate (KY11571) and our two samples isolated from the spiders formed a well-supported clade (Fig. 1). From a phylogenetic point of view, *A.tiankengensis* could not be distinguished from *A.araneogenus*, being inside the clade of the latter. Previous morphological observations revealed several differences in the characteristics between *A.araneogenus* and *A.tiankengensis* (Chen et al. 2018; Chen et al. 2022); however, our samples from different regions showed diversity of morphology in this study. The colony colour and the shape and size of the phialides and conidia of *A.araneogenus* and *A.tiankengensis*, amongst other morphological features, have been noted in our samples. There is reason to believe that distinguishing the two species is difficult because of the extensive overlap in morphological characteristics. Thus, we propose that *A.tiankengensis* is a synonym of *A.araneogenus*.

## ﻿Discussion

In this study, *Akanthomyces* comprised at least 36 species with a cosmopolitan distribution (Table 2). A collection of 31 isolates of unknown identity were shown to represent four known species, four new species and an undetermined species of *Akanthomyces*. The phylogenetic positions of the four known species were evaluated, based on phylogenetic inferences according to five loci, namely, ITS, nrLSU, *TEF*, *RPB1* and *RPB2*, including *A.araneogenus* from China, Thailand and Vietnam, *A.dipterigenus* and *A.waltergamsii* from China and *A.sulphureus* from Vietnam (see Table 2 and Fig. 1). The four new species, given the names *A.kunmingensis* and *A.subaraneicola* from China, *A.laosensis* from Laos and *A.pseudonoctuidarum* from Thailand, were recognised according to morphological characteristics and molecular data. The isolate YFCC 945 from China represented an unknown species in the genus *Akanthomyces*. Unfortunately, the isolate did not produce conidia or reproductive structures when grown on PDA and other media and they were, thus, tentatively treated as an undetermined species of *Akanthomyces*, pending further investigation.

**Table 2.** Species diversity, host/substrate and geographic distribution of *Akanthomyces* species.

Species	Host/Substrate	Know distribution	References
*Akanthomycesaculeatus*	Adult moth (Noctuidae; Sphingidae)	USA (Connecticut; Washington; Ontario); Brazil (Salvador); Amazon countries	Mains (1950); Sanjuan et al. (2014)
*Akanthomycesangustispora*	Coleopterous larva	USA (Nashville)	Mains (1950)
*Akanthomycesaranearum*	Spider (Araneae)	USA (North Carolina; Maine); Ceylon; Netherlands; Ghana (Begoro); China	Mains (1950); Samson and Evans (1974); Hsieh et al. (1997); Zare and Gams (2001)
*Akanthomycesaraneicola*	Spider (Araneae)	China (Guizhou)	Chen et al. (2019)
***Akanthomycesaraneogenus***	**Spider (Araneae)**	China (Guizhou; **Yunnan**); **Thailand (Chiang Mai); Vietnam (Nghe An)**	Chen et al. (2018); **This study**
*Akanthomycesaraneosus*	Spider (Araneae)	China (Guizhou)	Chen et al. (2022)
*Akanthomycesattenuatus*	*Cydiapomonella* (Lepidoptera, Tortricidae); leaf litter of *Acersaccharum*; *Symplocarpusfoetidus* (plants); *Astrocaryumsciophilum* (plants)	Poland; USA; Canada; French	Zare and Gams (2001); Ellsworth et al. (2013); Barthélemy et al. (2019)
*Akanthomycesclavata*	*Hapithusagitator* (Orthoptera, Gryllidae)	USA (Florida)	Mains (1950)
*Akanthomycescoccidioperitheciatus*	Spider (Araneae)	Japan	Kepler et al. (2017); Johnson et al. (2009)
***Akanthomycesdipterigenus***	Hemiptera: *Iceryapurchasi* (Coccidae); *Myzuspersicae* (Aphididae); *Macrosiphoniellasanborni* (Aphididae); *Citrusaphid* (Aphididae); **soil**	UK; Sri Lanka; Peru; **China (Yunnan)**	Kepler et al. (2017); Zare and Gams (2001); **This study**
*Akanthomycesfragilis*	Orthopterous larva	Trinidad; Guiana; Brazil	Mains (1950); Petch (1937)
*Akanthomycesgracilis*	Hymenoptera, Formicidae (*Paltothyreustarsatus*; *Platythyreaconradti*; *Polyrhachismilitaris*; *Polyrhachismonista*; *Polyrhachisdecemdentata*; *Camponotusbrutus*; *Oecophyllalonginoda*; *Crematogasterbequarti*; *Crematogasterclariventris*; *Macromischoidesinermis*; *Macromischoidesaculeatus*; *Dorylus* sp.); Coleoptera (beetle larvae, beetle imago); Lepidoptera larva; Hemiptera (Pyrrhocoridae; Cercopidae)	Ghana (Begoro); China (Guizhou)	Samson and Evans (1974); Liang et al. (2013)
*Akanthomycesjohnsonii*	Leaf and stem (*Arctium* sp., *Begonia* sp., *Coffea* sp., *Dianthus* sp., *Ipomoea* sp., *Kalanchoe* sp., *Lycopersicon* sp., *Peperomia* sp., and *Sargassum* sp.); often associated with species of *Botryosporium*	Ghana; Indonesia; Australia (Great Barrier Reef); UK; USA; Canada	Vincent et al. (1988)
*Akanthomyceskanyawimiae*	Spider (Araneae)	Thailand (Phetchabun; Chanthaburi)	Mongkolsamrit et al. (2018)
***Akanthomyceskunmingensis***	**Spider (Araneae)**	**China (Yunnan)**	**This study**
***Akanthomyceslaosensis***	**Adult moth (Lepidoptera, Noctuidae)**	**Laos (Oudomxay)**	**This study**
*Akanthomyceslecanii*	Hemiptera, Coccidae: *Pulvinariafloccifera*; *Coccusviridis*; scale insect. *Tetranychusurticae* (Acari: Tetranychidae); *Pistaciavera* (plants); *Ammophilaarenaria* (plants); *Dactylisglomerata* (plants); *Deschampsiaflexuosa* (plants); *Elymusfarctus* (plants); *Laretiaacaulis* (plants); *Pinussylvestris* (plants); *Shoreathumbuggaia* (plants); *Taxusbaccata* (plants)	W. Indies; Dominican Republic; Peru; Jamaica; USA; Sri Lanka; Indonesia; Turkey; China; Iran; Spain; Finland; Chile; Italy; Poland; India	Kepler et al. (2017); Zare and Gams (2001); Dash et al. (2018); Dolatabad et al. (2017); Nicoletti and Becchimanzi (2020)
*Akanthomyceslepidopterorum*	Pupa of Lepidoptera	China (Guizhou)	Chen et al. (2020b)
*Akanthomycesmuscarius*	*Trialeurodesvaporariorum* (Hemiptera, Aleyrodidae); *Brachycaudushelichrysi* (Hemiptera, Aphididae); *Cecidophyopsisribis* (Acari, Eriophyidae); *Cossuscossus* (Lepidoptera, Cossidae); *Zyginidiapullula* (Hemiptera, Cicadellidae); *Thripstabaci* (Thysanoptera, Thripidae); peat; contaminated pesticide solution; *Pteridiumaquilinum* (Pteridophyta); leaves of *Nypafruticans* (Plants); *Hemileiavastatrix* (Fungi); water from domestic supply; laboratory glyphosate solution; *Acercampestre* (plants); *Laurusnobilis* (plants); *Myrtuscommunis* (plants); *Nypafruticans* (plants); *Quercusrobur* (plants); *Prunuscerasus* (plants); *cabbage plants*	UK; Italy; New Caledonia; Thailand; New Zealand	Kepler et al. (2017); Zare and Gams (2001); Nicoletti and Becchimanzi (2020); Vinit et al. (2018); Aghdam and Fotouhifar 2017; Kuchár et al. (2019)
*Akanthomycesneoaraneogenus*	Spider (Araneae)	China (Guizhou)	Chen et al. (2017); Mains (1949)
*Akanthomycesneocoleopterorum*	Ladybug (Coleoptera)	China (Guizhou)	Chen et al. (2020a)
*Akanthomycesnoctuidarum*	Adult moth (Lepidoptera, Noctuidae)	Thailand (Narathiwat; Nakhon Ratchasima; Kamphaeng Phet)	Aini et al. (2020)
*Akanthomycespissodis*	Adult of *Pissodesstrobi* (Coleoptera, Curculionidae)	Canada	Cope and Leal (2005)
***Akanthomycespseudonoctuidarum***	**Adult moth (Lepidoptera, Noctuidae)**	**Thailand (Chiang Mai)**	**This study**
*Akanthomycespyralidarum*	Adult moth (Lepidoptera, Pyralidae)	Thailand (Kanchanaburi; Chiang Mai; Phetchabun)	Aini et al. (2020)
*Akanthomycesryukyuenis*	Spider (Araneae)	Japan	Kobayasi and Shimizu (1982)
*Akanthomycessabanensis*	*Pulvinariacaballeroramosae* (Hemiptera, Coccidae)	Colombia	Chiriví-Salomón et al. (2015)
***Akanthomycessubaraneicola***	**Spider (Araneae)**	**China (Hunan; Yunnan)**	**This study**
***Akanthomycessulphureus***	**Spider (Araneae)**	Thailand (Nakhon Ratchasima; Surat Thani); **Vietnam (Nghe An)**	Mongkolsamrit et al. (2018); **This study**
*Akanthomycesthailandicus*	Spider (Araneae)	Thailand (Chiang Mai)	Mongkolsamrit et al. (2018)
*Akanthomycestiankengensis*	Spider (Araneae)	China (Guizhou)	Chen et al. (2022)
*Akanthomycestortricidarum*	Adult moth (Lepidoptera, Tortricidae)	Thailand (Nakhon Ratchasima; Kamphaeng Phet)	Aini et al. (2020)
*Akanthomycestuberculatus* (= *A.pistillariaeformis*)	Adult moth (Lepidoptera); Hymenoptera, Formicidae; Hemiptera, Pyrrhocoridae	China (Zhejiang; Yunnan); Begoro; Trinidad	Mains (1950); Samson and Evans (1974); Liang et al. (2007)
*Akanthomycesuredinophilus*	Rust; decayed insect	Korea (Gangwon; North Chungcheong); China (Yunnan)	Park et al. (2016); Wei et al. (2018)
***Akanthomyceswaltergamsii***	**Spider (Araneae)**	Thailand (Saraburi; Naknon Ratchasima); **China (Yunnan)**	Mongkolsamrit et al. (2018); **This study**
*Akanthomyceszaquensis*	The stroma and the sclerotium of *Ophiocordycepssinensis* (Fungi)	China (Qinghai)	Wang et al. (2023a)

**Table 3.** Morphological comparison of *Akanthomyces* species.

Species	Perithecia (μm)	Asci (μm)	Part-spores (μm)	Synnemata (mm)	Conidiophores (μm)	Phialides (μm)	Conidia (μm)	References
*Akanthomycesaculeata*				Arising from various parts of the insect, terete, narrowing upwards, 1–8 × 0.1–0.5, yellowish		Subcylindrical or narrowly ellipsoidal, 6–16 × 2.5–4, narrowing above to an acute apex, terminated by a short sterigma up to 4 long	Broadly ellipsoidal or obovoid often acute at the lower end, 3–6 × 2–3	Mains (1950)
*Akanthomycesaranearum*				Arising from all parts of the host, cylindrical to clavate, 0.8–10 × 0.1–0.2, simple or occasionally slightly branched, brown		Obovoid or ellipsoidal 6–12 × 4–8, rounded above and abruptly narrowing into a short sterigma, asperulate	Narrowly obclavate often acute at the lower end, narrowing upwards, rounded or obtuse at the upper end, 8–14 × 1.5–3	Mains (1950)
*Akanthomycesaraneicola*				Synnemata not observed	Mononematous, with single phialide or whorls of two to six phialides or *Penicillium*-like from hyphae directly	Cylindrical, somewhat inflated base, 8.1–16.9 × 1.3–1.9, tapering to a thin neck	Mostly fusiform, 2.5–5.0 × 1.3–1.9	Chen et al. (2019)
*Akanthomycesaraneogenus*				Occasionally several white synnemata arise from all parts of the host	Mononematous or synnematous, 21.6–48 × 1.2–2.2, *Penicillium*-like from hyphae directly	Cylindrical, somewhat inflated base, 4.3–17.3 × 0.9–3.1, tapering to a thin neck	Globose, 1.3–2.4 in diam, or ellipsoidal, 2.1–3.3 × 1.1–1.6	Chen et al. (2018)
*Akanthomycesaraneosus*				Synnemata not observed	Erect conidiophores usually arose from the aerial hyphae	Solitary or in groups of two, 16.9–18.1 × 1.3–1.9 with a cylindrical basal portion and tapered into a short, distinct neck	Fusiform, 3.1–5.0 × 1.0–1.8	Chen et al. (2022)
*Akanthomycesangustispora*				Arising from the body and head of the host, simple or branched, 8–13 × 0.2–0.6, flesh coloured		Oblong or narrowly ellipsoidal, 6–14 × 3–4, narrowing above into an acute apex terminated by a short sterigma	Narrowly clavate, 4.5–6 × 1.2–1.4	Mains (1950)
*Akanthomycesattenuatus*						9–15.5 × 1–2	Cylindrical with attenuate base, occasionally 2-celled, 4.5–6.5 × 1.5–2.0	Zare and Gams (2001); Kepler et al. (2017)
*Akanthomycesclavata*				Numerous, arising from various parts of the host, light brown, clavate, 0.5–2.0 × 0.06–0.25		Subcylindrical, 17.1–21.4 × 2.8–4.3, narrowing above to acute apices, terminated by short sterigmata	Ellipsoidal to oblong, 4.5–8.5 × 2.1–2.5	Mains (1950)
*Akanthomycesdipterigenus*						20–40 × 1.2–2.7, tapering towards the apex	Ellipsoidal to oblong-oval, 5.0–10.5 × 1.5–2.5	Zare and Gams (2001); Kepler et al. (2017)
*Akanthomycesfragilis*				Numerous arising from all parts of the host, clavate, 0.7–1.5 × 0.03–0.09		Subcylindrical to narrowly clavate, 7–10 × 2.5–3, verrucose in the upper portions	Subcylindrical, somewhat narrowed and rounded at the ends, 6.5–9 × 1.5	Mains (1950)
*Akanthomycesgracilis*				Arising from the natural body openings and intersegmental and appendage joints, usually white to yellow-brown, cylindrical, 0.7–3 × 0.1–0.5		Cylindrical basal part tapering to a slender neck, 7–10 × 1.5–2.5	Ellipsoidal to fusiform, 2.5–3 × 1–1.6	Samson and Evans (1974)
*Akanthomycesjohnsonii*				Gregarious, white, 0.4–4 tall, with a stipe 0.025–0.1 wide, subulate to cylindrical	Unbranched or with metulae arising at right angles to the stipe hyphae, 4–6 × 2–3	10–20 long, ellipsoidal to cylindrical body 2.5–4 wide, tapering into a narrow neck 3–5 × 1–1.5	Broadly fusoid with more or less truncate poles with minute frills, 3–4 × (l–)1.5–2	Vincent et al. (1988)
*Akanthomyceskanyawimiae*				Up to 1.5 long, up to 0.4 wide, covered by dense white to cream mycelia	Erect, verticillate with phialides in whorls of two to five	(8–)9–12(–15) × 2–3, with cylindrical basal portion, tapering into a long neck, (2–)3–5.5(–7) × 1–1.5	Cylindrical to ellipsoidal, (2–)2.5–3.5(–5) × (1.5–)2(–3)	Mongkolsamrit et al. (2018)
***Akanthomyceskunmingensis***				**Cream to light yellow, erect, irregularly branched**	**Cylindrical, solitary, sometimes verticillate, with phialides in whorls of four to five 4.3–9.5 × 1.2–2.0**	**Cylindrical, somewhat inflated base, 6.2–29.4 × 1.1–2.5**	**Ellipsoidal to long oval, 1.9–3.5 × 1.1–1.8**	**This study**
***Akanthomyceslaosensis***				**Arising at the head and in the middle of the host body, white, up to 15.6 long, 0.6–1.3 wide, feathery to clavate with acute or blunt end**	**Monophialidic, produced along the synnemata or solitary on hyphae in culture**	**Cylindrical, 11.5–30.0 × 2.0–4.2**	**Cylindrical or long oval, 4.1–9.8 × 2.3–4.2**	**This study**
*Akanthomyceslecanii*	Ovoid, 350–650 × 200–375	200–350 × 3.5–4				Relatively short, 11–20 (–30) × 1.3–1.8, aculeate and strongly tapering	Typically short-ellipsoidal, 2.5–3.5 (–4.2) × 1–1.5	Kepler et al. (2017); Zare and Gams (2001); Shrestha et al. (2019)
*Akanthomyceslepidopterorum*				Synnemata not observed	Mononematous, with single phialide or two phialides	Cylindrical, somewhat inflated base, 12.7–25.8 × 1.4–1.7, tapering to a thin neck	Mostly cylindrical, 3.5–5.6 × 1.4–2.1, forming mostly globose heads	Chen et al. (2020b)
*Akanthomycesmuscarius*						(15–)20–35 × 1.0–1.7	Ellipsoidal to subcylindrical, (2–)2.5–5.5(–6) × 1–1.5(–1.8)	Kepler et al. (2017); Zare and Gams (2001)
*Akanthomycesneoaraneogenus*				Synnemata not observed	Moderately branched, with (1–)2–6 (–8) phialides	30–64 × 1.1–3.2	Forming mostly globose heads, cylindrical, 3.2–8.6 × 1.3–1.6	Chen et al. (2017); Mains (1949)
*Akanthomycesneocoleopterorum*				Synnemata not observed	Mononematous, with single phialide or whorls of two to five phialides, or *Verticillium*-like from hyphae directly	Cylindrical, somewhat inflated base, 19.9–29.6 × 1.6–2.0, tapering to a thin neck	Mostly cylindrical, 3.3–6.6 × 1.5–1.8	Chen et al. (2020a)
*Akanthomycesnoctuidarum*	Ovoid, (530–)623–993(–1000) × (290–)308–413(–425)	(170–)196–423(–550) × (2–)2.7–3.8(–4)	(6–)7–10.7(–13) × 1	Arising from moth body and wing veins, white to cream, erect, cylindrical to clavate, (650–)668–1191(–1500) × (50–)53.4–102(–120) µm	Monophialidic or polyphialidic	Cylindrical with papillate end, hyaline, (5–)6.8–9(–10) × (1.8–)2–2.4(–3)	Cylindrical with round end, (3–)3.5–4.5(–6) × 1	Aini et al. (2020)
*Akanthomycespissodis*				Synnemata not observed			Cylindrical to ovoid or oval, 4–9.2 × 1.6–2.4	Cope and Leal (2005)
***Akanthomycespseudonoctuidarum***				**Arising from moth body, cream to light yellow, erect, cylindrical to clavate, 0.8–2 × 0.12–0.35**	**Cylindrical, solitary, 6.5–13.8 × 1.8–3.6**	**Cylindrical with papillate end, 6.8–26.0 × 2.1–3.6**	**Ellipsoidal to long oval, 2.6–6.4 × 1.5–2.2**	**This study**
*Akanthomycespyralidarum*	Ovoid to obpyriform, (290–)342–580(–650) × (150–)186–291(–340)	(170–)222–329(–360) × (2–)2.5–.3(–4)	(5–)5.9–9.4(–12) × 1	Synnemata not observed	Not observed	Not observed	Not observed	Aini et al. (2020)
*Akanthomycesryukyuenis*	Pyriformia, 570–630 × 170–250	5 wide, cap 3 wide	1 × 1–4					Kobayasi and Shimizu (1982)
*Akanthomycessabanensis*				Synnemata not observed	Generally arising from submerged hyphae, moderately branched	Solitary or in whorls of 2–4, 13–19 long, from 1.0–2.0 gradually tapering to 0.5–1.0	Ellipsoidal to ovoid, usually straight, 3.5–4.5 × 1.5–2.0	Chiriví-Salomón et al. (2015); Kepler et al. (2017)
*Akanthomycessulphureus*	Narrowly ovoid, (650–)676(–680) × (240–)324.5(–330)	Up to 500 long, 2–3 wide	(300–)336(–450) × 1–1.5	Synnemata not observed	Erect, verticillate with phialides in whorls of two to three	(10–)16(–20) × 2–2.5, with a cylindrical basal portion, tapering into a thin neck, 1 × 0.5	Cylindrical to ellipsoidal, (4–)4.5–5.5(–6) × 2–3	Mongkolsamrit et al. (2018)
***Akanthomycessubaraneicola***				**Synnemata not observed**	**Cylindrical, solitary or verticillate with phialides in whorls of two to five, 6.5–12.3 × 1.6–3.5**	**Cylindrical, somewhat inflated base, 12.1–38.2 × 1.3–3.2**	**Ellipsoidal to long oval, 3.0–5.4 × 1.8–3.4**	**This study**
*Akanthomycesthailandicus*	Narrowly ovoid, (700–)752–838(–850) × (300–)305–375(–400)	Up to 550 long, 5–7 wide	4–6 × 1–1.5	Synnemata not observed	Erect, forming verticillate branches with solitary phialides	(12–)13.5–21(–30) × 1–2, awl-shaped, *lecanicillium*-like	Cylindrical to ellipsoidal (3–)4–6(–7) × 1.5–2	Mongkolsamrit et al. (2018)
*Akanthomycestiankengensis*				Synnemata not observed	Erect, usually aring from the aerial hyphae	Solitary or in groups of two, 13.9–17.1 × 1.1– 1.6 with a cylindrical basal portion and tapering into a short, distinct neck	Fusiform, 2.3–3.0 × 1.5–2.3	Chen et al. (2022)
*Akanthomycestortricidarum*				Long synnemata aring at the head and in the middle of the host body, up to 5 long, 0.12–0.15 wide, cylindrical to clavate, short synnemata aring on moth body, wings and legs, (197–)200–267(–300) × (15–)17.7–31.6(40–)µm, white to cream	Monophialidic or polyphialidic	Long synnemata: (5–)6–8(–10) × (1.8–)2–2.7(–3), short synnemata: (5–)6.2–8.3(–10) × (1.8–)2–2.5(–3), cylindrical to ellipsoidal with papillate end	Fusoid, long synnemata: (1–)2.5–3(–3.2) × (0.8–)1–1.4(–2), short synnemata: (1–)1.8–2.7(–3) × 1–2	Aini et al. (2020)
*Akanthomycestuberculatus* (= *A.pistillariaeformis*)	Narrowly ovoid or conoid, 420–900 × 180–370	300–600 × 4–5	2–6 × 0.5–1	Arising from all parts of the moths, clavate, 0.4–1.0 long, the stipe 0.025–0.05 thick		Subcylindrical, 6–10 × 2–3, narrowing above into an acute apex terminated by a short sterigma 2–3 long	Fusoid to subcylindrical narrowing at the ends, 2.5–5 × 1–1.5	Mains (1950)
*Akanthomycesuredinophilus*				Synnemata not observed		Produced singly or in whorls of up to 3–4(–5) on prostrate hyphae, 20–60 × 1–2.5(–3)	Cylindrical, oblong, or ellipsoid, 3–9 × 1.8–3	Park et al. (2016)
*Akanthomyceswaltergamsii*				Arising on legs of spider, erect, up to 1.5 long, 0.1–0.12 wide	Usually forming verticillate branches with phialides in whorls of two to five	(10–)16(–22) × (1–)1.5(–2), with cylindrical to ellipsoidal basal portion, tapering into a thin neck, 1–3 × 1	Ellipsoidal or fusiform, (2–)3.5(–4) × 2–3	Mongkolsamrit et al. (2018)
*Akanthomyceszaquensis*				Synnemata not observed		8.0–40.0 long, rarely over 100, 0.6–1.2 at the base, tapering to about 0.4 at the tips	Long-ellipsoidal to almost cylindrical, (1.5–)3.0–6.0(–7.0) × 0.5–1.2(–1.5)	Wang et al. (2023a)

The highest species diversity of *Akanthomyces* occurred in subtropical and tropical regions, especially in China and Southeast Asia (see Table 2). Based on our update, there are at least 17 *Akanthomyces* species in China and Yunnan Province has the most. There is also high species diversity of *Akanthomyces* in Southeast Asia, where more than 11 species have been recorded (Table 2). Thailand, Vietnam and Laos are located in tropical regions with extremely rich biodiversity in Southeast Asia. The forests exhibit a significant variety of plant and animal life attributed to the tropical monsoon climate, characterised by high temperatures and rainfall (Lao et al. 2021). These have created a favourable environment for the development of arthropod-pathogenic fungi, including *Akanthomyces* spp.

*Akanthomyces* species inhabit diverse hosts/substrates that range from eight orders of Arthropoda, namely, Acari, Araneae, Coleoptera, Hemiptera, Hymenoptera, Lepidoptera, Orthoptera and Thysanoptera, to plants, other fungi, peat, water and rusts (see Table 2). Amongst the hosts of *Akanthomyces*, Araneae and Lepidoptera are the two major orders. Our study also found that the majority of *Akanthomyces* species are spider pathogens or adult moth entomopathogens, with the exception of a few other entomopathogens and generalists that have a remarkably broad host/substrate range (Table 2 and Fig. 1). In this study, we identified an extension of the host/substrate range to also include soil, as shown in Fig. 1. The family Cordycipitaceae has been shown to evolve from an ancestor which is ecologically versatile and most probably inhabit the soil/environment and diversified into groups of entomopathogens and mycoparasites (Sung et al. 2007; Kepler et al. 2017; Wang et al. 2020; Zhou et al. 2022). *Akanthomyces* have been principally shown to be arthropod-pathogenic fungi in this study. The fact that *Akanthomyces* can be found in soil might suggest some kind of convergence/reversion.

Due to the difficulty of isolation and the limitation of cultivation conditions, studies on the development and application of *Akanthomyces* species are still currently limited. As generalists that have a remarkably broad host/substrate range, *A.gracilis* and *A.muscarius* have a high potential for interspecific transmission and biological control of pest insects (Samson and Evans 1974; Zare and Gams 2001; Kuchár et al. 2019; Nicoletti and Becchimanzi 2020). *Akanthomyceslecanii* is an effective mycoparasite of several rust fungi, green mould and fungi causing root rot diseases (*Pythiumultimum*), as well as of several powdery mildew pathogens and it is receiving increasing attention as a versatile biocontrol agent of a number of plant pathogens (Benhamou and Brodeur 2001). The members of the genus *Akanthomyces* contain species ranging from specialists with very narrow host ranges to generalists that attack a wide range of arthropods and they might be used as an ideal model system for research on fungal arthropod pathology and fungal-pathogen speciation and host adaptation (Hu et al. 2014). Coleopterans, lepidopterans and spiders are the major host groups of arthropod-pathogenic fungi within Hypocreales (Shrestha et al. 2019). The findings indicate that the majority of the hosts of *Akanthomyces* are distributed in lepidopterans and spiders, with a few in coleopterans (see Table 2). These arthropod-pathogenic fungi with special nutritional preferences are more likely to produce numerous distinctive bioactive compounds. It is hoped that this study will generate continued interest amongst mycologists, arachnologists and related experts and researchers to use such fungal resources through in vitro growth and extraction of useful bio-active secondary metabolites (extrolites).

Fungal species diversity and their host/substrate associations are important aspects of fungal ecology. A strong taxonomic basis that is dependent on advances in nucleic acid sequence technology is one of the main fundamental needs in fungal ecology (Zhang et al. 2021) and is even crucial to studies on species diversity and their host–substrate associations. However, it is regrettable that a growing number of researchers have relied heavily on molecular biology techniques to the complete exclusion of fungal isolation and characterisation utilising classical methods (Walker et al. 2019; Zhang et al. 2021). Although fungal research has entered the molecular era, phenotypic and culture-based studies are still an invaluable tool for fungal biology and ecology exploration (Walker et al. 2019). In addition to molecular data, morphological and ecological characteristics have a pivotal role in taxonomy and phylogenetic identification of fungi. In our work, we surveyed the literature to the greatest extent possible, combined that with the results of those obtained by morphological methods (optical microscope and electron microscope) in our study, to list and compare the morphological characteristics of 35 *Akanthomyces* species (Table 3). The morphological comparison revealed obvious differences in the size of ascospores and asci, morphology of the synnemata, conidiogenous structures and conidial shape and size, although the morphological features generally overlapped. Our statistics showed that at least 20 *Akanthomyces* species are specialists with narrow host ranges and they are either spider pathogens or adult moth entomopathogens (Table 2). They cause mortality of spiders and adult moths by nature. The cadavers are usually found attached to the underside of leaves or on tree trunks, barks, decaying logs, branches, grass, leaf litter and forest floors (Shrestha et al. 2019). These ecological characteristics are phylogenetically informative for distinguishing species of *Akanthomyces* and they contribute to the timely discovery of new *Akanthomyces* species in nature.

## Supplementary Material

XML Treatment for Akanthomyces kunmingensis

XML Treatment for Akanthomyces laosensis

XML Treatment for Akanthomyces pseudonoctuidarum

XML Treatment for Akanthomyces subaraneicola

XML Treatment for Akanthomyces araneogenus

## ﻿Additional information

### Author ORCIDs

Yao Wang https://orcid.org/0000-0002-1262-6700

Hong Yu https://orcid.org/0000-0002-2149-5714

### Data availability

All of the data that support the findings of this study are available in the main text or Supplementary Information.

## Supplementary materials

Supplementary material 1 Supplementary information Data type docx Explanation note **fig. S1.** Phylogenetic relationships among the genus *Akanthomyces* and its allies in Cordycipitaceae based on Bayesian inference (BI) and maximum likelihood (ML) analyses of a five-locus (ITS, nrLSU, *TEF*, *RPB1*, and *RPB2*) dataset. **fig. S2.** Phylogenetic tree of *Akanthomyces* based on Maximum Likelihood (IQ-TREE) analysis from the ITS sequences. Statistical support values (≥70%) are shown at the nodes for ML boostrap support. **fig. S3.** Phylogenetic tree of *Akanthomyces* based on Maximum Likelihood (IQ-TREE) analysis from the nrLSU sequences. **fig. S4.** Phylogenetic tree of *Akanthomyces* based on Maximum Likelihood (IQ-TREE) analysis from the *TEF* sequences. **fig. S5.** Phylogenetic tree of *Akanthomyces* based on Maximum Likelihood (IQ-TREE) analysis from the *RPB1* sequences. **fig. S6.** Phylogenetic tree of *Akanthomyces* based on Maximum Likelihood (IQ-TREE) analysis from the *RPB2* sequences. https://binary.pensoft.net/file/967825This dataset is made available under the Open Database License (http://opendatacommons.org/licenses/odbl/1.0/). The Open Database License (ODbL) is a license agreement intended to allow users to freely share, modify, and use this Dataset while maintaining this same freedom for others, provided that the original source and author(s) are credited.Yao Wang, Zhi-Qin Wang, Run Luo, Sisommay Souvanhnachit, Chinnapan Thanarut, Van-Minh Dao, Hong Yu

## References

[B1] Aghdam SA Fotouhifar KB (2017) Introduction of some endophytic fungi of sour cherry trees (*Prunuscerasus*) in Iran. Rostaniha 18: 77–94.

[B2] Aini AN Mongkolsamrit S Wijanarka W Thanakitpipattana D Luangsaard JJ Budiharjo A (2020) Diversity of *Akanthomyces* on moths (Lepidoptera) in Thailand. MycoKeys 71: 1–22. 10.3897/mycokeys.71.55126 32831550 PMC7410849

[B3] Barthélemy M Elie N Pellissier L Wolfender JL Stien D Touboul D Eparvier V (2019) Structural identification of antibacterial lipids from Amazonian palm tree endophytes through the molecular network approach. International Journal of Molecular Sciences 20(8): 2006–2018. 10.3390/ijms20082006 31022840 PMC6514718

[B4] Benhamou N Brodeur J (2001) Pre-inoculation of Ri T-DNA transformed cucumber roots with the mycoparasite, *Verticilliumlecanii*, induces host defense reactions against *Pythiumultimum* infection. Physiological and Molecular Plant Pathology 58(3): 133–146. 10.1006/pmpp.2001.0322

[B5] Bischoff JF Rehner SA Humber RA (2006) *Metarhiziumfrigidum* sp. nov.: A cryptic species of *M.anisopliae* and a member of the *M.flavoviride* complex. Mycologia 98(5): 737–745. 10.1080/15572536.2006.11832645 17256577

[B6] Boudier E (1885) Note sur un nouveau genre et quelques nouvelles especes des Pyrenomycetes. Revue Mycologique Toulouse 7: 224–227.

[B7] Chen WH Han YF Liang ZQ Jin DC (2017) *Lecanicilliumaraneogenum* sp. nov., a new araneogenous fungus. Phytotaxa 305(1): 29–34. 10.11646/phytotaxa.305.1.4

[B8] Chen WH Liu C Han YF Liang JD Liang ZQ (2018) *Akanthomycesaraneogenum*, a new *Isaria*-like araneogenous species. Phytotaxa 379(1): 66–72. 10.11646/phytotaxa.379.1.6

[B9] Chen WH Liu C Han YF Liang JD Tian WY Liang ZQ (2019) *Akanthomycesaraneicola*, a new araneogenous species from Southwest China. Phytotaxa 409(4): 227–232. 10.11646/phytotaxa.409.4.5

[B10] Chen WH Han YF Liang JD Liang ZQ (2020a) *Akanthomycesneocoleopterorum*, a new verticillium-like species. Phytotaxa 432: 119–124. 10.11646/phytotaxa.432.2.2

[B11] Chen WH Han YF Liang JD Liang ZQ (2020b) *Akanthomyceslepidopterorum*, a new lecanicillium-like species. Phytotaxa 459: 117–123. 10.11646/phytotaxa.459.2.3

[B12] Chen WH Liang JD Ren XX Zhao JH Han YF Liang ZQ (2022) Species diversity of *Cordyceps*-like fungi in the Tiankeng karst region of China. Microbiology Spectrum 10(5): e01975–e22. 10.1128/spectrum.01975-22 PMC9603550 36094103

[B13] Chen WH Liang JD Ren XX Zhao JH Han YF (2023) Study on species diversity of *Akanthomyces* (Cordycipitaceae, Hypocreales) in the Jinyun Mountains, Chongqing, China. MycoKeys 98: 299–315. 10.3897/mycokeys.98.106415 37547126 PMC10403762

[B14] Chiriví-Salomón JS Danies G Restrepo S Sanjuan T (2015) *Lecanicilliumsabanense* sp. nov. (Cordycipitaceae) a new fungal entomopathogen of coccids. Phytotaxa 234(1): 63–74. 10.11646/phytotaxa.234.1.4

[B15] Cope HH Leal I (2005) A new species of *Lecanicillium* isolated from the white pine weevil, *Pissodesstrobi*. Mycotaxon 94: 331–340.

[B16] Darriba D Taboada GL Doallo R Posada D (2012) jModelTest 2: More models, new heuristics and parallel computing. Nature Methods 9(8): e772. 10.1038/nmeth.2109 PMC4594756 22847109

[B17] Dash CK Bamisile BS Ravindran K Qasim M Lin Y Islam SU Hussain M Wang L (2018) Endophytic entomopathogenic fungi enhance the growth of *Phaseolusvulgaris* L. (Fabaceae) and negatively affect the development and reproduction of *Tetranychusurticae* Koch (Acari: Tetranychidae). Microbial Pathogenesis 125: 385–392. 10.1016/j.micpath.2018.09.044 30290267

[B18] Dolatabad HK Javan-Nikkhah M Shier WT (2017) Evaluation of antifungal, phosphate solubilisation, and siderophore and chitinase release activities of endophytic fungi from *Pistaciavera*. Mycological Progress 16(8): 777–790. 10.1007/s11557-017-1315-z

[B19] Ellsworth KT Clark TN Gray CA Johnson JA (2013) Isolation and bioassay screening of medicinal plant endophytes from eastern Canada. Canadian Journal of Microbiology 59(11): 761–765. 10.1139/cjm-2013-0639 24206359

[B20] Gams W Zare R (2001) A revision of Verticilliumsect.Prostrata. III. Generic classification. Nova Hedwigia 72(3–4): 329–337. 10.1127/nova.hedwigia/72/2001/329

[B21] Hsieh LS Tzean SS Wu WJ (1997) The genus *Akanthomyces* on spiders from Taiwan. Mycologia 89(2): 319–324. 10.1080/00275514.1997.12026788

[B22] Hu X Xiao G Zheng P Shang Y Su Y Zhang X Liu X Zhan S St Leger RJ Wang C (2014) Trajectory and genomic determinants of fungal-pathogen speciation and host adaptation. Proceedings of the National Academy of Sciences of the United States of America 111: 16796–16801. 10.1073/pnas.1412662111 25368161 PMC4250126

[B23] Johnson D Sung GH Hywel-Jones NL Luangsa-Ard JJ Bischoff JF Kepler RM Spatafora JW (2009) Systematics and evolution of the genus *Torrubiella* (Hypocreales, Ascomycota). Mycological Research 113(3): 279–289. 10.1016/j.mycres.2008.09.008 18938242

[B24] Kepler RM Sung GH Ban S Nakagiri A Chen MJ Huang B Li Z Spatafora JW (2012) New teleomorph combinations in the entomopathogenic genus *Metacordyceps*. Mycologia 104(1): 182–197. 10.3852/11-070 22067304

[B25] Kepler RM Luangsa-ard JJ Hywel-Jones NL Quandt CA Sung GH Rehner SA Aime MC Henkel TW Sanjuan T Zare R Chen MJ Li ZZ Rossman AY Spatafora JW Shrestha B (2017) A phylogenetically-based nomenclature for Cordycipitaceae (Hypocreales). IMA Fungus 8(2): 335–353. 10.5598/imafungus.2017.08.02.08 29242779 PMC5729716

[B26] Kobayasi Y Shimizu D (1982) *Cordyceps* species from Japan 5. Bulletin of the National Science Museum Series B 8: 111–123.

[B27] Kuchár M Glare TR Hampton JG Dickie IA Christey MC (2019) Virulence of the plant-associated endophytic fungus *Lecanicilliummuscarium* to diamondback moth larvae. New Zealand Plant Protection 72: 253–259. 10.30843/nzpp.2019.72.257

[B28] Lanfear R Calcott B Ho SYW Guindon S (2012) Partitionfinder: Combined selection of partitioning schemes and substitution models for phylogenetic analyses. Molecular Biology and Evolution 29(6): 1695–1701. 10.1093/molbev/mss020 22319168

[B29] Lao TD Le TAH Truong NB (2021) Morphological and genetic characteristics of the novel entomopathogenic fungus *Ophiocordycepslangbianensis* (Ophiocordycipitaceae, Hypocreales) from Lang Biang Biosphere Reserve, Vietnam. Scientific Reports 11(1): e1412. 10.1038/s41598-020-78265-7 PMC7809459 33446667

[B30] Lebert H (1858) Ueber einige neue oder unvollkommen gekannte Krankheiten der Insekten, welche durch Entwicklung niederer Pflanzen im lebenden Körper enstehen. Zeitschrift für Wissenschaftliche Zoologie 9: 439–453.

[B31] Liang ZQ Liu AY Liu ZY (2007) *Cordyceps*. Flora Fungorum sinicorum (Vol. 32). Science Press, Beijing.

[B32] Liang ZQ Chen WH Han YF Zou X (2013) A combined identification of morphological traits and DELTA system to *Akanthomycesgracilis* from China. Journal of Fungal Research 11: 242–245.

[B33] Liu ZY Liang ZQ Whalley AJS Yao YJ Liu AY (2001) *Cordycepsbrittlebankisoides*, a new pathogen of grubs and its anamorph, Metarhiziumanisopliaevar.majus. Journal of Invertebrate Pathology 78(3): 178–182. 10.1006/jipa.2001.5039 11812122

[B34] Mains EB (1949) New species of *Torrubiella*, *Hirsutella* and *Gibellula*. Mycologia 41(3): 303–310. 10.1080/00275514.1949.12017774

[B35] Mains EB (1950) Entomogenous species of *Akanthomyces*, *Hymenostilbe* and *Insecticola* in North America. Mycologia 42(4): 566–589. 10.1080/00275514.1950.12017861

[B36] Manfrino R Gutierrez A Diez del Valle F Schuster C Ben Gharsa H López Lastra C Leclerque A (2022) First description of *Akanthomycesuredinophilus* comb. nov. from Hemipteran insects in America. Diversity 14(12): e1118. 10.3390/d14121118

[B37] Minh BQ Schmidt HA Chernomor O Schrempf D Woodhams MD Von Haeseler A Lanfear R (2020) IQ-TREE 2: New models and efficient methods for phylogenetic inference in the genomic era. Molecular Biology and Evolution 37(5): 1530–1534. 10.1093/molbev/msaa015 32011700 PMC7182206

[B38] Mongkolsamrit S Noisripoom W Thanakitpipattana D Wutikhun T Spatafora JW Luangsa-Ard J (2018) Disentangling cryptic species with *Isaria*-like morphs in Cordycipitaceae. Mycologia 110(1): 230–257. 10.1080/00275514.2018.1446651 29863995

[B39] Nicoletti R Becchimanzi A (2020) Endophytism of *Lecanicillium* and *Akanthomyces*. Agriculture 10(6): 1–16. 10.3390/agriculture10060205

[B40] Park MJ Hong SB Shin HD (2016) *Lecanicilliumuredinophilum* sp. nov. associated with rust fungi from Korea. Mycotaxon 130(4): 997–1005. 10.5248/130.997

[B41] Petch T (1937) Notes on entomogenous fungi. Transactions of the British Mycological Society 21(1–2): 34–67. 10.1016/S0007-1536(37)80005-4

[B42] Rehner SA Buckley E (2005) A *Beauveria* phylogeny inferred from nuclear ITS and EF1-a sequences: Evidence for cryptic diversification and links to *Cordycepsteleomorphs*. Mycologia 97(1): 84–98. 10.3852/mycologia.97.1.84 16389960

[B43] Rehner SA Samuels GJ (1994) Taxonomy and phylogeny of *Gliocladium* analysed from nuclear large subunit ribosomal DNA sequences. Mycological Research 98(6): 625–634. 10.1016/S0953-7562(09)80409-7

[B44] Ronquist F Teslenko M van der Mark P Ayres DL Darling A Höhna S Larget B Liu L Suchard MA Huelsenbeck JP (2012) MrBayes 3.2: Efficient Bayesian phylogenetic inference and model choice across a large model space. Systematic Biology 61(3): 539–542. 10.1093/sysbio/sys029 22357727 PMC3329765

[B45] Samson RA Evans HC (1974) Notes on entomogenous fungi from Ghana II. The genus *Akanthomyces*. Acta Botanica Neerlandica 23(1): 28–35. 10.1111/j.1438-8677.1974.tb00913.x

[B46] Sanjuan T Tabima J Restrepo S Læssøe T Spatafora JW Franco-Molano AE (2014) Entomopathogens of Amazonian stick insects and locusts are members of the *Beauveria* species complex (*Cordyceps* sensu stricto). Mycologia 106(2): 260–275. 10.3852/13-020 24782494

[B47] Shrestha B Kubátová A Tanaka E Oh J Yoon DH Sung JM Sung GH (2019) Spider-pathogenic fungi within Hypocreales (Ascomycota): Their current nomenclature, diversity, and distribution. Mycological Progress 18(8): 983–1003. 10.1007/s11557-019-01512-3

[B48] Simon C Frati F Beckenbach A Crespi B Liu H Flook P (1994) Evolution, weighting, and phylogenetic utility of mitochondrial gene sequences and a compilation of conserved polymerase chain reaction primers. Annals of the Entomological Society of America 87(6): 651–701. 10.1093/aesa/87.6.651

[B49] Stamatakis A Hoover P Rougemont J (2008) A rapid bootstrap algorithm for the RAxML web servers. Systematic Biology 57(5): 758–771. 10.1080/10635150802429642 18853362

[B50] Sung GH Hywel-Jones NL Sung JM Luangsa-ard JJ Shrestha B Spatafora JW (2007) Phylogenetic classification of *Cordyceps* and the clavicipitaceous fungi. Studies in Mycology 57: 5–59. 10.3114/sim.2007.57.01 18490993 PMC2104736

[B51] Swofford DL (2002) PAUP*. Phylogenetic analysis using parsimony (*and other methods), version 4.0b10. Sinauer Associates, Sunderland.

[B52] Tamura K Stecher G Peterson D Filipski A Kumar S (2013) MEGA6: Molecular evolutionary genetics analysis version 6.0. Molecular Biology and Evolution 30(12): 2725–2729. 10.1093/molbev/mst197 24132122 PMC3840312

[B53] Vilgalys R Hester M (1990) Rapid genetic identification and mapping of enzymatically amplified ribosomal DNA from several *Cyptococcus* species. Journal of Bacteriology 172(8): 4238–4246. 10.1128/jb.172.8.4238-4246.1990 2376561 PMC213247

[B54] Vincent MA Seifert KA Samson RA (1988) *Akanthomycesjohnsonii*, a saprophytic synnematous hyphomycete. Mycologia 80(5): 685–688. 10.1080/00275514.1988.12025601

[B55] Vinit K Doilom M Wanasinghe DN Bhat DJ Brahmanage RS Jeewon R Xiao Y Hyde KD (2018) Phylogenetic placement of *Akanthomycesmuscarius*, a new endophyte record from *Nypafruticans* in Thailand. Current Research in Environmental & Applied Mycology 8(3): 404–417. 10.5943/cream/8/3/10

[B56] Walker LM Cedeño-Sanchez M Carbonero F Herre EA Turner BL Wright SJ Stephenson SL (2019) The response of litter-associated Myxomycetes to long-term nutrient addition in a lowland tropical forest. The Journal of Eukaryotic Microbiology 66(5): 757–770. 10.1111/jeu.12724 30793409

[B57] Wang Y Wang YR Han YF Liang ZQ (2015) A new thermotolerant species of *Taifanglania*. Junwu Xuebao 34: 345–349. 10.13346/j.mycosystema.140136

[B58] Wang YB Wang Y Fan Q Duan DE Zhang GD Dai RQ Dai YD Zeng WB Chen ZH Li DD Tang DX Xu ZH Sun T Nguyen TT Tran NL Dao VM Zhang CM Huang LD Liu YJ Zhang XM Yang DR Sanjuan T Liu XZ Yang ZL Yu H (2020) Multigene phylogeny of the family Cordycipitaceae (Hypocreales): New taxa and the new systematic position of the Chinese cordycipitoid fungus *Paecilomyceshepiali*. Fungal Diversity 103(1): 1–46. 10.1007/s13225-020-00457-3

[B59] Wang ZQ Wang Y Dong QY Fan Q Dao VM Yu H (2022) Morphological and phylogenetic characterization reveals five new species of *Samsoniella* (Cordycipitaceae, Hypocreales). Journal of Fungi 8(7): e747. 10.3390/jof8070747 PMC9321185 35887502

[B60] Wang YH Wang WJ Wang K Dong CH Hao JR Kirk PM Yao YJ (2023a) *Akanthomyceszaquensis* (Cordycipitaceae, Hypocreales), a new species isolated from both the stroma and the sclerotium of *Ophiocordycepssinensis* in Qinghai, China. Phytotaxa 579(3): 198–208. 10.11646/phytotaxa.579.3.5

[B61] Wang Y Tang DX Luo R Wang YB Thanarut C Dao VM Yu H (2023b) Phylogeny and systematics of the genus *Clonostachys*. Frontiers in Microbiology 14: e1117753. 10.3389/fmicb.2023.1117753 PMC10020229 36937310

[B62] Wei DP Wanasinghe DN Chaiwat TA Hyde KD (2018) *Lecanicilliumuredinophilum* known from rusts, also occurs on animal hosts with chitinous bodies. Asian Journal of Mycology 1(1): 63–73. 10.5943/ajom/1/1/5

[B63] White TJ Bruns T Lee S Taylor JW (1990) Amplification and direct sequencing of fungal ribosomal RNA genes for phylogenetics. PCR protocols, 315–322. 10.1016/B978-0-12-372180-8.50042-1

[B64] Zare R Gams W (2001) A revision of VerticilliumsectionProstrata. IV. The genera *Lecanicillium* and *Simplicillium* gen. nov. Nova Hedwigia 73(1–2): 1–50. 10.1127/nova.hedwigia/73/2001/1

[B65] Zhang ZY Shao QY Li X Chen WH Liang JD Han YF Huang JZ Liang ZQ (2021) Culturable fungi from urban soils in China I: Description of 10 new taxa. Microbiology Spectrum 9(2): e00867–e21. 10.1128/Spectrum.00867-21 PMC8510251 34612666

[B66] Zhou YM Zhi JR Qu JJ Zou X (2022) Estimated divergence times of *Lecanicillium* in the family Cordycipitaceae provide insights into the attribution of *Lecanicillium*. Frontiers in Microbiology 13: e859886. 10.3389/fmicb.2022.859886 PMC9121009 35602068

