# Supplementary material for: ﻿Species diversity and major host/substrate associations of the genus Akanthomyces (Hypocreales, Cordycipitaceae)
Source: MycoKeys. 2024 Jan 15;101:113–41. doi: 10.3897/mycokeys.101.109751 (PMC10806914; doi:10.3897/mycokeys.101.109751)
Supplement: Supplementary material 1 — Supplementary information [file mycokeys-101-113-s001.docx]

**
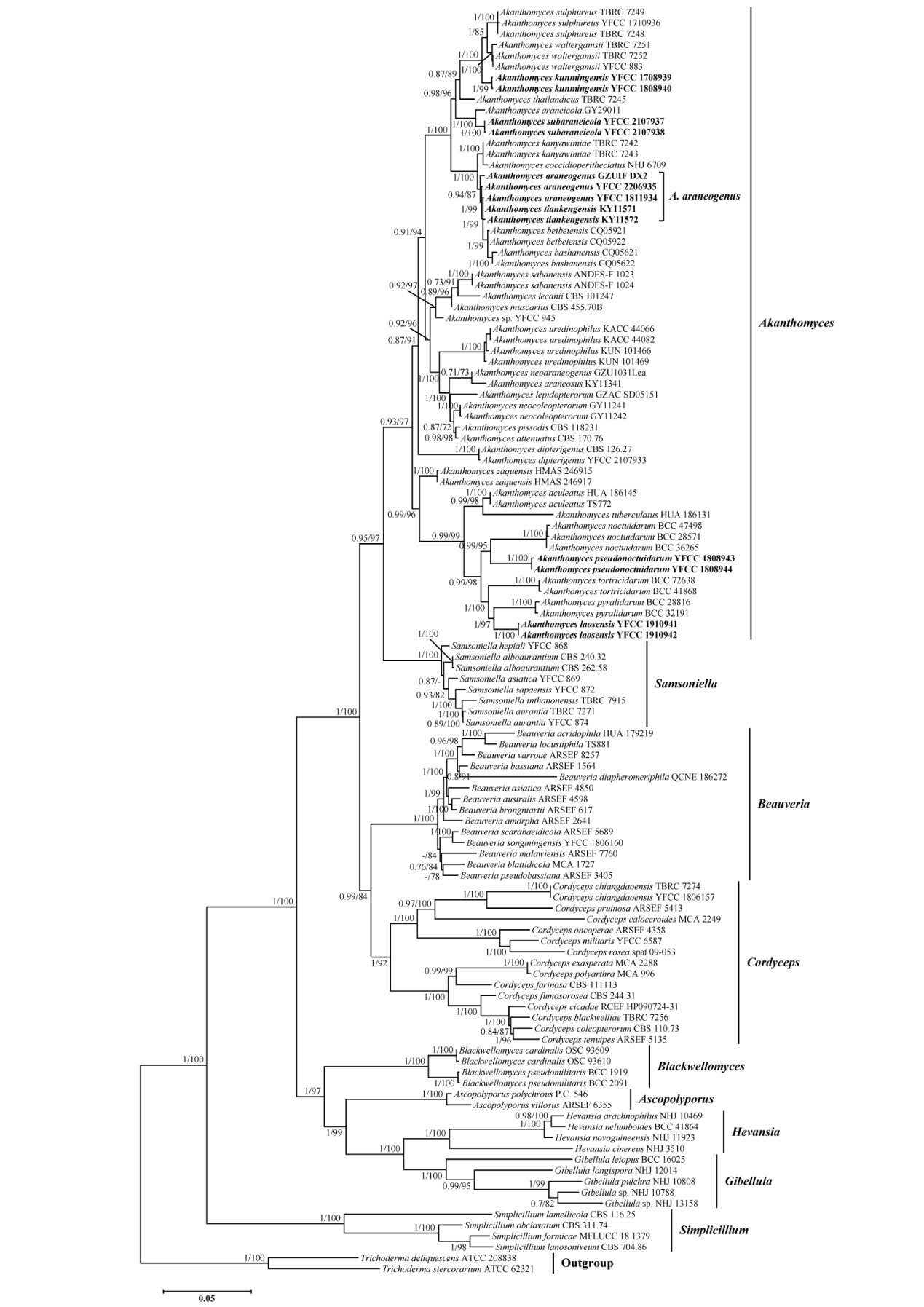
**

**Figure S1.** Phylogenetic relationships among the genus *Akanthomyces* and its allies in Cordycipitaceae based on Bayesian inference (BI) and maximum likelihood (ML) analyses of a five-locus (ITS, nr*LSU*, *TEF*, *RPB1*, and *RPB2*) dataset. No significant differences in topology are observed between BI and ML (IQ-TREE) phylogenies. Statistical support values (≥ 0.7/70%) are shown at the nodes for BI posterior probabilities/ ML bootstrap support. Isolates in bold type are those analyzed in this study.


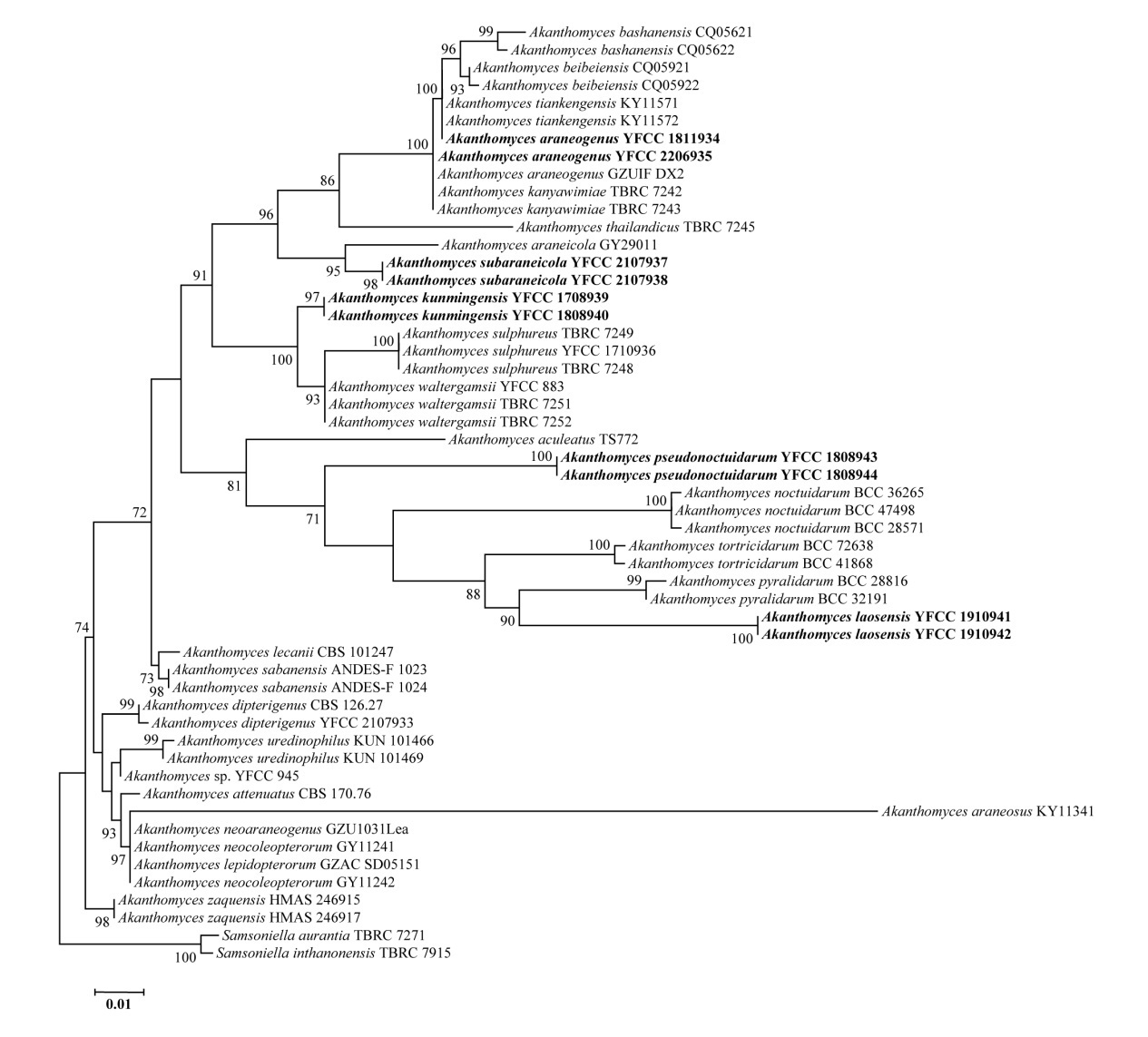


**Figure S2.** Phylogenetic tree of *Akanthomyces* based on Maximum Likelihood (IQ-TREE) analysis from the ITS sequences. Statistical support values (≥70%) are shown at the nodes for ML boostrap support. Isolates in bold type are those analyzed in this study.


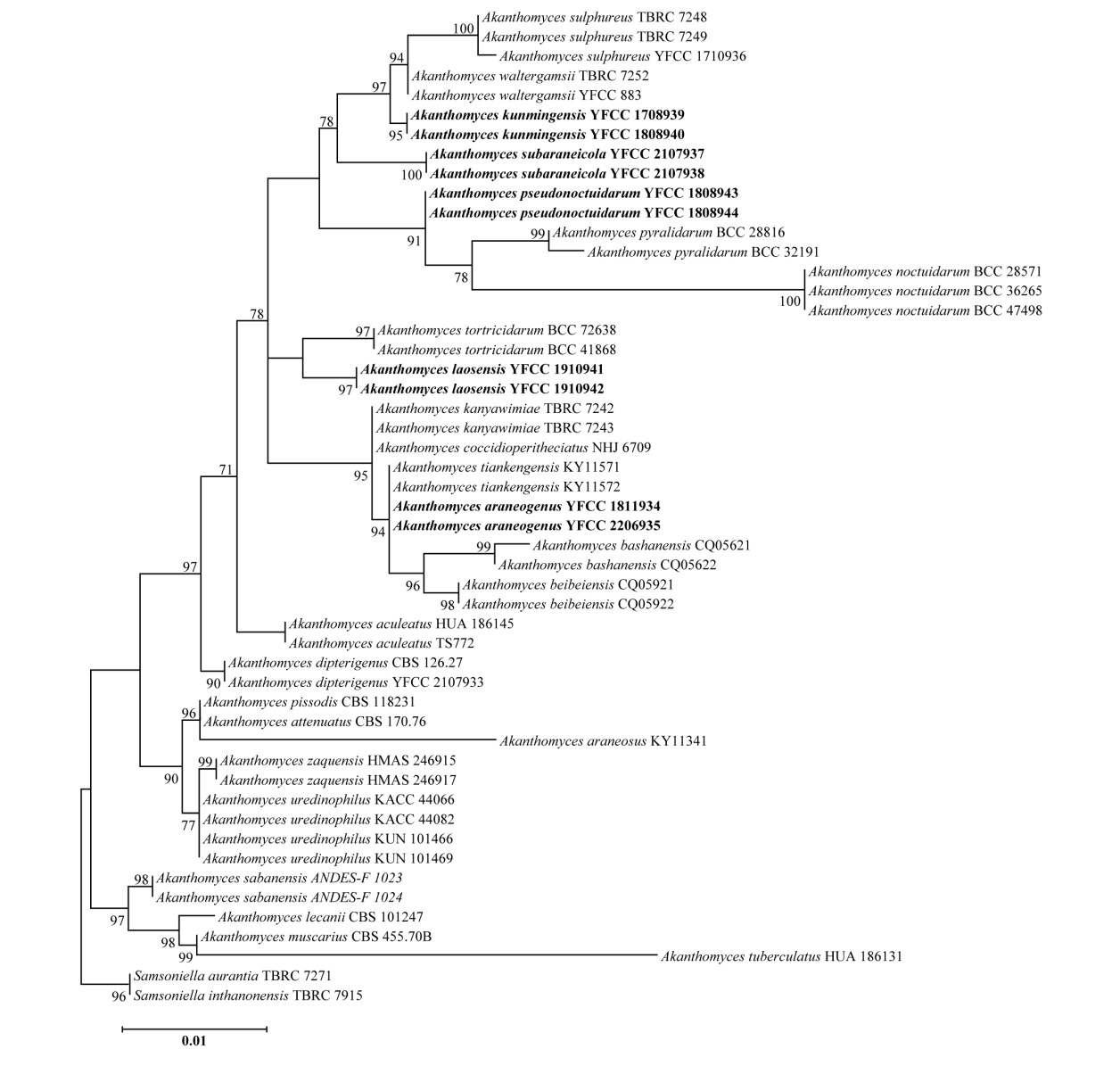


**Figure S3.** Phylogenetic tree of *Akanthomyces* based on Maximum Likelihood (IQ-TREE) analysis from the nr*LSU* sequences. Statistical support values (≥70%) are shown at the nodes for ML boostrap support. Isolates in bold type are those analyzed in this study.


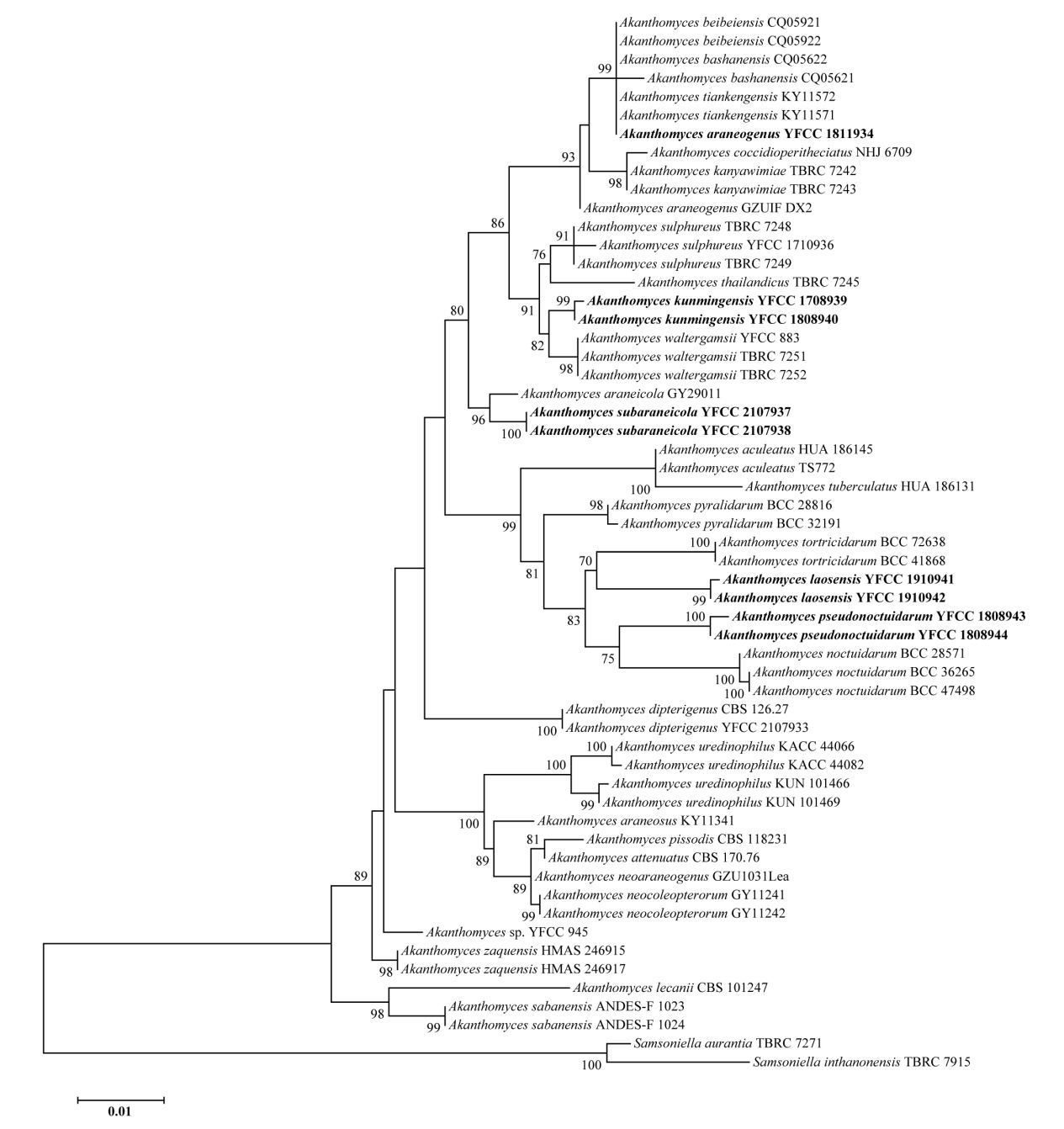


**Figure S4.** Phylogenetic tree of *Akanthomyces* based on Maximum Likelihood (IQ-TREE) analysis from the *TEF* sequences. Statistical support values (≥70%) are shown at the nodes for ML boostrap support. Isolates in bold type are those analyzed in this study.


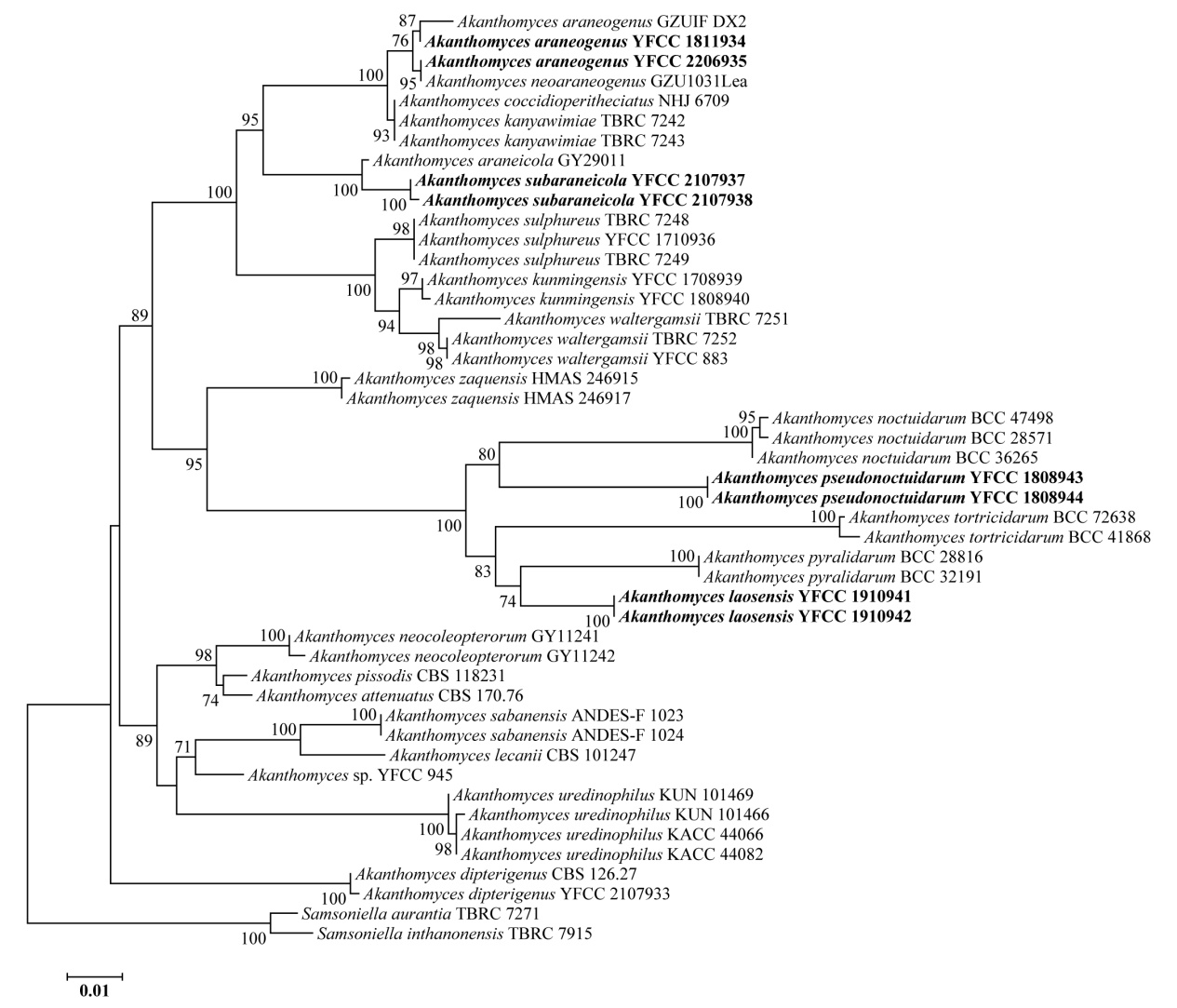


**Figure S5.** Phylogenetic tree of *Akanthomyces* based on Maximum Likelihood (IQ-TREE) analysis from the *RPB1* sequences. Statistical support values (≥70%) are shown at the nodes for ML boostrap support. Isolates in bold type are those analyzed in this study.


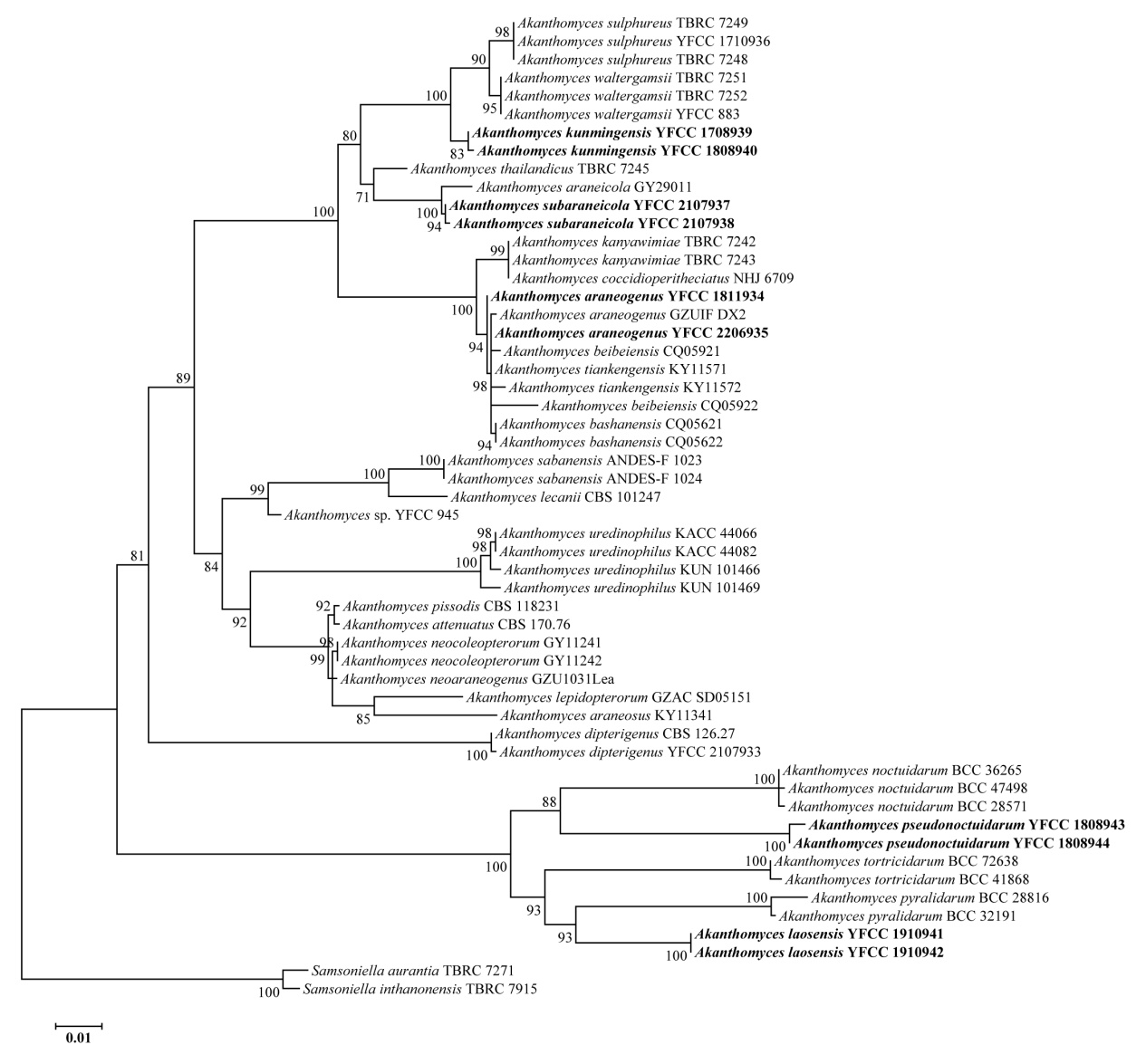


**Figure S6.** Phylogenetic tree of *Akanthomyces* based on Maximum Likelihood (IQ-TREE) analysis from the *RPB2* sequences. Statistical support values (≥70%) are shown at the nodes for ML boostrap support. Isolates in bold type are those analyzed in this study.
